# Decorating polymer beads with 10^14^ inorganic-organic [2]rotaxanes as shown by spin counting

**DOI:** 10.1038/s42004-022-00689-1

**Published:** 2022-06-20

**Authors:** Deepak Asthana, Dean Thomas, Selena J. Lockyer, Adam Brookfield, Grigore A. Timco, Iñigo J. Vitorica-Yrezabal, George F. S. Whitehead, Eric J. L. McInnes, David Collison, David A. Leigh, Richard E. P. Winpenny

**Affiliations:** 1 Department of Chemistry, The University of Manchester, Oxford Road, Manchester, M13 9Pl UK; 2 Department of Chemistry, Ashoka University, Sonipat, Haryana India

**Keywords:** Interlocked molecules, Coordination chemistry

## Abstract

Polymer beads have been used as the core of magnetic particles for around twenty years. Here we report studies to attach polymetallic complexes to polymer beads for the first time, producing beads of around 115 microns diameter that are attached to 10^14^ hybrid inorganic-organic [2]rotaxanes. The bead is then formally a [10^14^] rotaxane. The number of complexes attached is counted by EPR spectroscopy after including TEMPO radicals within the thread of the hybrid [2]rotaxanes.

## Abstract (web summary)

Polymer beads are used in the core of magnetic particles, and beads functionalised with paramagnetic molecules are promising as agents for dynamic nuclear polarization. Here, the authors use conventional click chemistry to decorate a polymer bead with 10^14^ [2]rotaxanes containing paramagnetic {Cr_7_Ni} rings.

## Introduction

Since 1999 polymer beads have been used as the core of magnetic particles, with Caruso et al. showing that magnetite could be grown on the exterior of polystyrene^1^. This hugely influential work involved use of a polyelectrolyte attached to the polystyrene core before attachment of Fe_3_O_4_ nanoparticles. Similar approaches have been used to make core-shell structures with other inorganic materials. ZnS has been grown around polystyrene as a route to photonic crystals^2^ and metal-organic framework shells have been grown as a route to particles that can be used to adsorb pollutants^3,4^.

Beads functionalised with paramagnetic molecules have been used in catalysis^5^ and studied as agents for dynamic nuclear polarisation (DNP)^6^. Further functionality has been introduced by blending these properties on or in polymer hosts, and such magnetic polymer beads have been exploited in many areas, including sensing, imaging and separations^7^. Radical-bead functionalisation has been used to probe the macromolecular structures of the beads and their swelling on solvation^8^.

We wondered if it was possible to attach polymetallic compounds to a polystyrene bead. This could then be developed in two distinct directions: the attached compound could be decomposed to give a core-shell particle with great control of the metal shell, and perhaps allow formation of different metal-shells than are accessible from synthesis from simple precursors. Secondly, decorating a bead with a very large number of highly paramagnetic molecules could lead to a use in DNP or as a contrast agent or even as a ferrofluid. However, before speculating on possible applications we needed to establish that the chemistry itself is possible. This is purpose of this report.

**Fig. 1:**
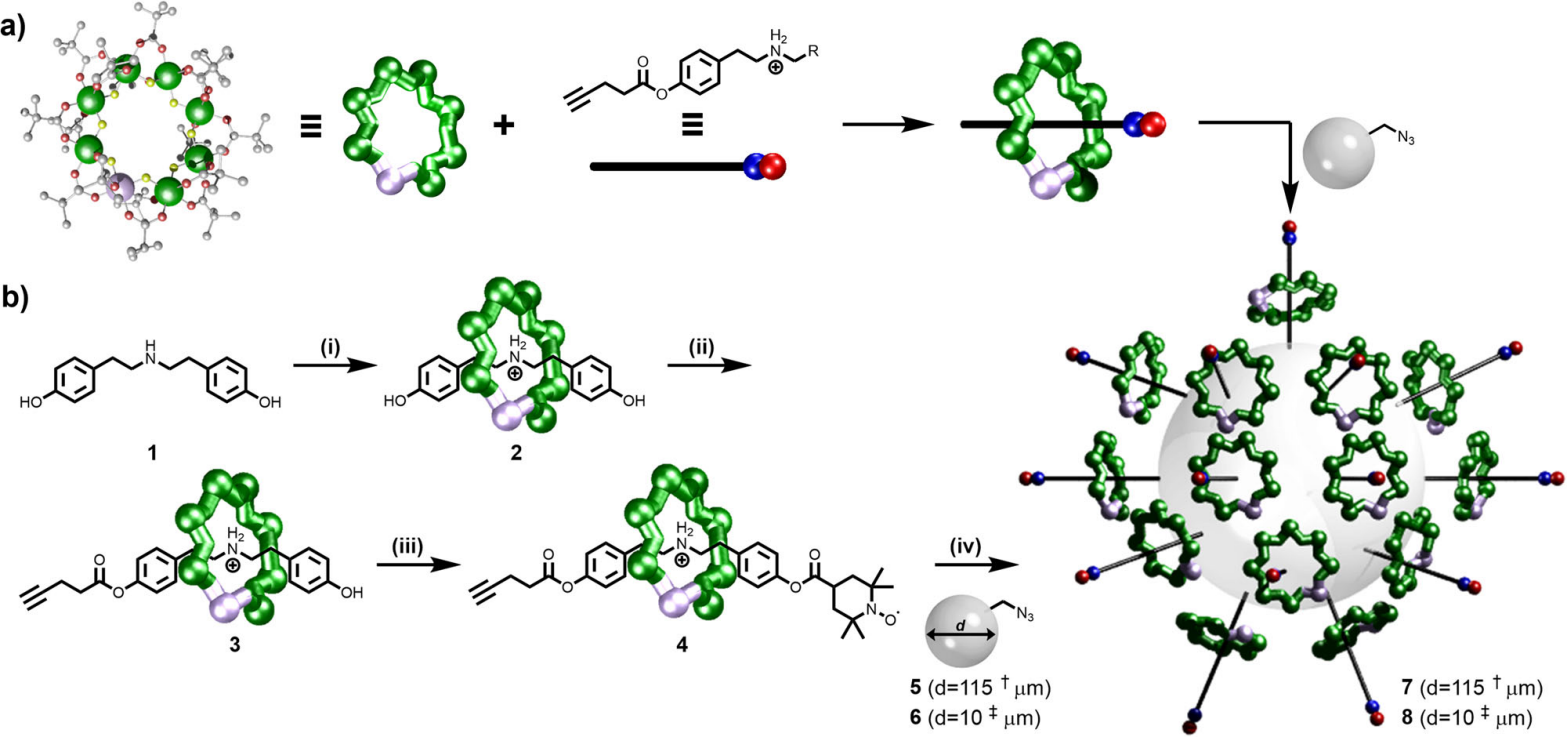
Synthesis of a decorated bead. **a** General overview. **b** Reaction conditions: (i) Pivalic acid, 2NiCO_3_·3Ni(OH)_2_·4H_2_O, CrF_3_·4H_2_O, 140 °C, 24 h; (ii) Pentynoic acid, DCC, DMAP, THF, 50 °C, 16 h; (iii) 4-Carboxy-TEMPO, DCC, DMAP, CH_2_Cl_2_, RT, 48 h. (iv) Azide-terminated polystyrene beads, [Cu(MeCN)_4_]PF_6_, CH_2_Cl_2_:DMF (3:2), RT, 72 h. † - 115 ± 35 μm. ‡ - 10 ± 1 μm. TEMPO 2,2,6,6-Tetramethylpiperidine 1-oxyl, DCC N,N′-Dicyclohexylcarbodiimide, DMAP 4-Dimethylaminopyridine, THF tetrahydrofuran.

Well established chemistry was chosen. To form the link from the bead to the paramagnetic molecule we use conventional copper-catalysed azide-alkyne cycloaddition (CuAAC) click chemistry^9^. To enable this to work we functionalised commercially available polystyrene microspheres with pendant azides (Fig. 1). As the polymetallic unit we use {Cr_7_Ni} rings that we have studied over a period of years^10^. Here, we use the rings as part of a hybrid inorganic–organic [2]rotaxane^11,12^ and then perform the click chemistry at one end of a carefully designed thread. We find that the chemistry is sufficiently flexible to allow several different [2]rotaxanes to be clicked onto the bead, including [2]rotaxanes that contain organic radicals in addition to the {Cr_7_Ni} rings. This has allowed us to perform an experiment where we count the spins from the organic radicals and that allows us to calculate that 10^14^ [2]rotaxanes are attached to the bead. As the bead is a cross-linked polymer, it means that each bead formally corresponds to a [10^14^]rotaxane.

We note that the first reported [2]rotaxane was prepared by repetitive statistical threading-and-capping of a polymer-supported macrocycle^13^.

## Results

### Synthesis

The approach is summarised in Fig. 1 and is based on the {Cr_7_Ni} rings forming around a secondary ammonium cation template, while the exterior of the ring is covered by sixteen pivalate groups, giving [(R_2_NH_2_)Cr_7_NiF_8_(^*t*^BuCO_2_)_16_]. Full details are given in the Methods Section and the Supplementary methods sections S1, S3 and S4.

The thread (HOC_6_H_4_CH_2_CH_2_)_2_NH **1** is formed from a primary amine and an alkyl bromide, each bearing a terminal phenolic group, and is the neutral precursor to the ammonium template. Choice of the distance between the ammonium and phenol unit is important. If the distance is too short, the phenol will be too sterically hindered for further functionalisation. If the distance is too long, the phenol is esterified by reaction with the pivalic acid used during the formation of the ring^12^. Here, we aimed for an intermediate distance, but a small amount of esterified product is always obtained when **1** is used to form [2]rotaxanes. This ability to control the reactivity of the phenol is important as it allows difunctionalisation of the thread.

Rotaxane **2** was prepared by reacting **1** with hydrated chromium(III) fluoride and basic nickel carbonate in pivalic acid (See Methods section for preparative details) and was purified by column chromatography. X-ray quality single crystals of **2** were grown from acetone by slow evaporation. The crystal structure of **2** shows the inorganic–organic [2]rotaxane with the axle terminated by phenols.

To introduce a terminal alkyne for CuAAC click chemistry, rotaxane **2** was reacted with 4-pentynoic acid under Steglich esterification conditions to yield **3**. A second esterification reaction with 4-carboxy-TEMPO gave **4**. Formation of rotaxane **4** was confirmed by electrospray mass spectrometry (ESI-MS), elemental analysis and single-crystal X-ray crystallography.

Rotaxane **4** was reacted with azide-functionalised polymer beads **5** or **6** (Fig. 1, see Methods section for preparative details) in a heterogeneous click reaction. After reaction with the rotaxanes, the initially colourless beads turned deep green giving the decorated beads **7** and **8**. To remove any unreacted [2]rotaxane and the catalyst, the beads were then washed repeatedly with CH_3_CN, CH_3_OH and CH_2_Cl_2_ following recommended methods^14^.

Product formation was monitored by FT-IR spectroscopy. The IR absorption peak for an azide group around 2090 cm^−1^ was used as a reference. After reaction with [2]rotaxane **4**, a significant decrease in the intensity of the azide band was observed (see SI, section 4). We attribute the small, residual azide band in the IR spectrum to azide functionalities present in inner, inaccessible regions of the bead^15^.

To estimate the number of solvent-accessible sites we made the following assumptions. Firstly, that the specification given for the beads is accurate (https://www.sigmaaldrich.com/GB/en/product/mm/855011, http://www.rapp-polymere.com/index.php?id=893&currency=968, http://www.rapp-polymere.com/index.php?id=1219&currency=498), and that the polymer bead is spherical. Secondly, that the density of accessible sites is uniform across the polymer bead, and that the number of accessible surface sites is not limited to the surface of the bead as there is a degree of swelling and ‘depth’ in which molecules can react at sites further inside the sphere. We then have to estimate a lower bound depth and we chose 0.5 µm. Thirdly, that the surface volume can be determined from the volume of two spheres of different diameters and that the density of polymer sites is uniform at the surface. Given these assumptions and assuming that all azide sites within 0.5 μm of the surface react, the number of rotaxanes on a single bead of diameter 115 µm, **5**, was estimated to be at least 2 × 10^13^ and for the smaller beads, **6**, we estimate that 8 × 10^10^rotaxanes could be attached (see SI, section 2, Fig. S11). The upper bound of these calculations, if all sites within the polymer beads react was 7 × 10^14^ rotaxanes for **5** and 3 × 10^11^ rotaxanes for **6**. We also assume that the azide formation and click reactions proceed quantitatively^16,17^.

### X-ray crystal structures

**Fig. 2:**
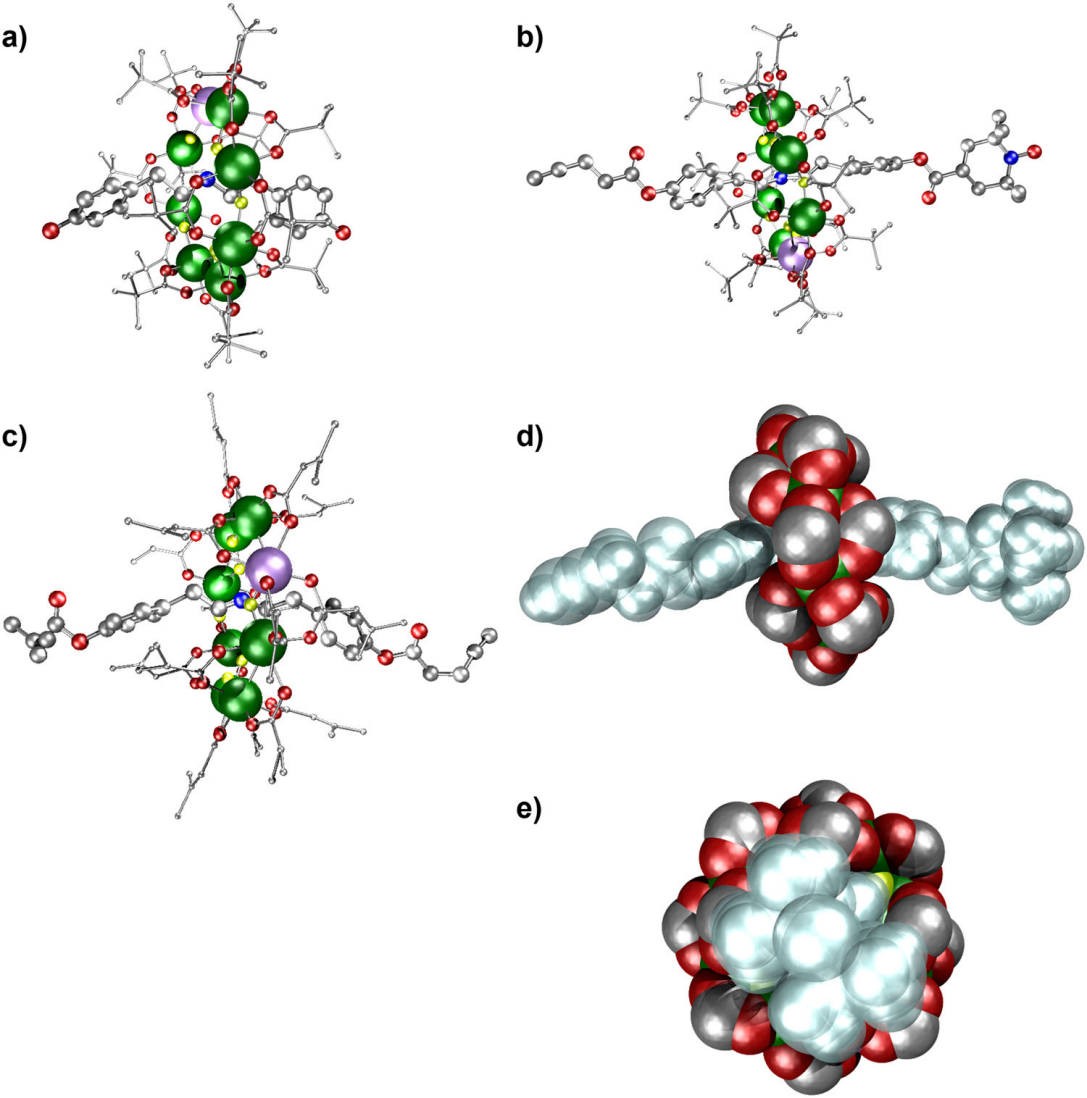
X-Ray crystal structures of compounds **2**, **4** and **10**. Ball and stick representations: **a** **2**; **b** **4**; **c** **10**. Space-filling representations of **4**: **d** into the edge of the {Cr_7_Ni} ring; **e** perpendicular to the Cr_7_Ni} ring. Colours in **a**, **b** and **c**: Cr-green, Ni-violet, F-yellow, O-red, N-blue and C-grey. Colours in **d** and **f**: thread shown in silver with pivalate groups on rings excluded for clarity. H-atoms excluded for clarity throughout.

Both **2** and **4** crystallise in monoclinic space groups: *P*2_1_/*c* and *C*2/*c* respectively (Table S1). The structures are shown in Fig. 2. In both structures the eight metals are arranged in an octagon, and the single divalent metal site is not identified by the crystallography but it is disordered about the octagon. Each metal…metal edge is bridged by two carboxylates outside the ring, and by a single fluoride, which lies within a cavity. The eight fluorides therefore provide hydrogen bond acceptor sites, and these form H-bonds to the protonated nitrogen of the threads. The metal sites are all six-coordinate, bound to four O- and two F-donors. The metal–ligand distances are very similar to those found in previous {Cr_7_Ni} rings^10^. For attachment to a bead the key metric is the distance from the terminal C-atom of the alkyne to the N-atom at the ring centre and it is around 1.36 nm, which allows the CuAAC click reaction to proceed.

### Electron paramagnetic resonance (EPR) spectroscopy and spin counting

**Fig. 3:**
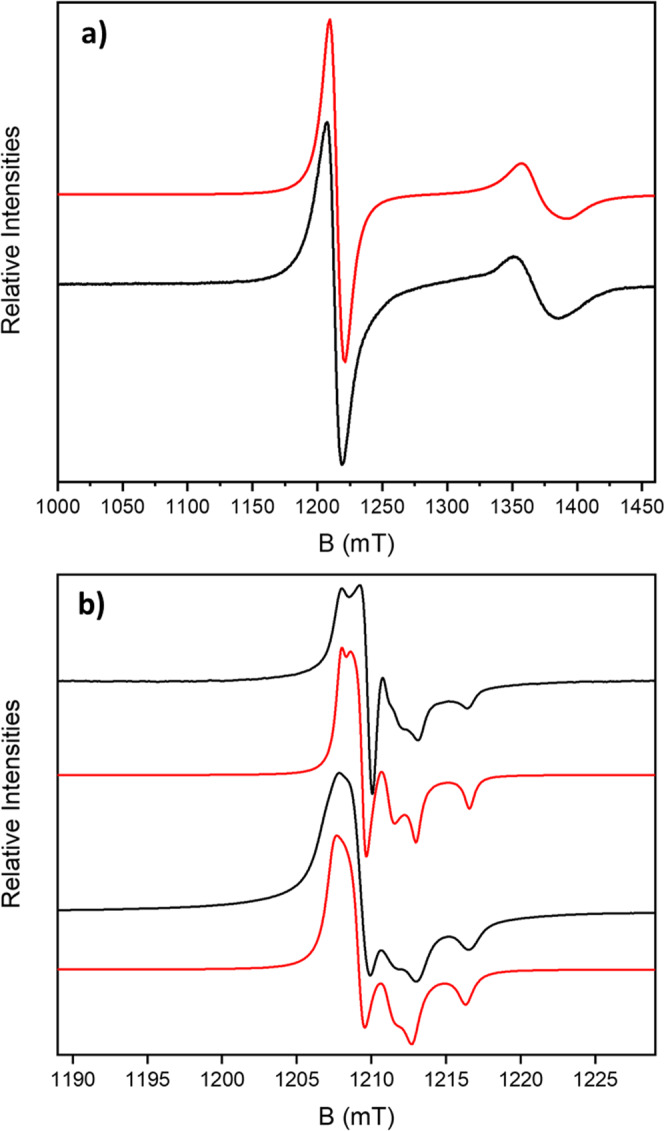
CW Q-Band (ca. 34 GHz) EPR spectra. **a** Powder sample of **4** at 5 K (black) and simulation (red), with the nitroxide and {Cr_7_Ni} resonances. **b** Multiple bead samples of **7** at 280 K (black top) and 5 K (black bottom), with their respective simulations (red), focused at the nitroxide signal. The experimental frequencies are **a** 34.032921 GHz and **b** 34.033889 GHz (280 K) and 34.040610 GHz (5 K).

Continuous wave (CW) Q-Band (ca. 34 GHz) EPR spectra of **4** and **7** were recorded at 5 K. There is a resonance at *g* = 1.78, which is only observed at low temperature, which arises from the *S* = ½ ground state of the {Cr_7_Ni} ring (Fig. 3 and S19)^18^. This signal is not perturbed between the rotaxane **4** and bead **7**. All spectra were simulated using parameters given in Table 1.

**Table 1:** EPR simulation parameters for compounds **4**, **7** and **8**.

	T/ K	NO˙ *g* values	^N^A_z_/MHz	Line width NO˙/mT	{Cr_7_Ni} *g* values	Line width {Cr_7_Ni}/mT
**4**	5	2.0106, 2.0060, 2.0030	100	10	1.788, 1.788, 1.748	20
**7**	5	2.0106, 2.0060, 2.0030	100	1.2	1.775, 1.775, 1.737	20
**7**	280	2.0097, 2.0057, 2.0026	100	0.8	N/A	N/A
**8**	5	2.0106, 2.0060, 2.0030	100	5	1.775, 1.775, 1.737	15

Rotaxane **4** and beads **7** and **8** contain TEMPO radicals terminating the thread, and the EPR spectra show resonances for the nitroxides that are observed at all temperatures (Fig. 3). In powdered [2]rotaxane **4**, the nitroxide spectrum is broad due to intermolecular interactions, whilst for bead **7** the signal is sharper, with partial resolution of the ^14^N hyperfine coupling, consistent with greater dilution of the rings on the bead surface. There are several previous examples of rotaxanes that involve TEMPO radicals^19–21^.

The nitroxide signal at RT for **7** makes it possible to quantify the number of nitroxides, and hence the number of threaded rings, on an individual bead, although the values approach the limit of EPR sensitivity^22^. Performing the calculation at lower temperatures is far more demanding, especially due to fluctuations in temperature that could influence the intensity of the signal. It is far easier to perform this at the nitroxide resonance than at the {Cr_7_Ni} resonance as the nitroxide resonance is visible at room temperature (Fig. 3b) and is far sharper than the broad resonance due to the heterometallic ring.

EPR signal intensity calibration curves were constructed from measurements on the [2]rotaxane **4** in toluene solution at room temperature (Fig. S19a, see SI, section 5), using twelve concentrations between 0.005 and 0.20 mM (Figs. S21 and S22). However, in repeated calibrations we found a discontinuity in the signal intensity as a function of concentration at around 0.07 mM, hence we have restricted spin counting on the beads to calibrant concentrations between 0.005 and 0.06 mM.

**Fig. 4:**
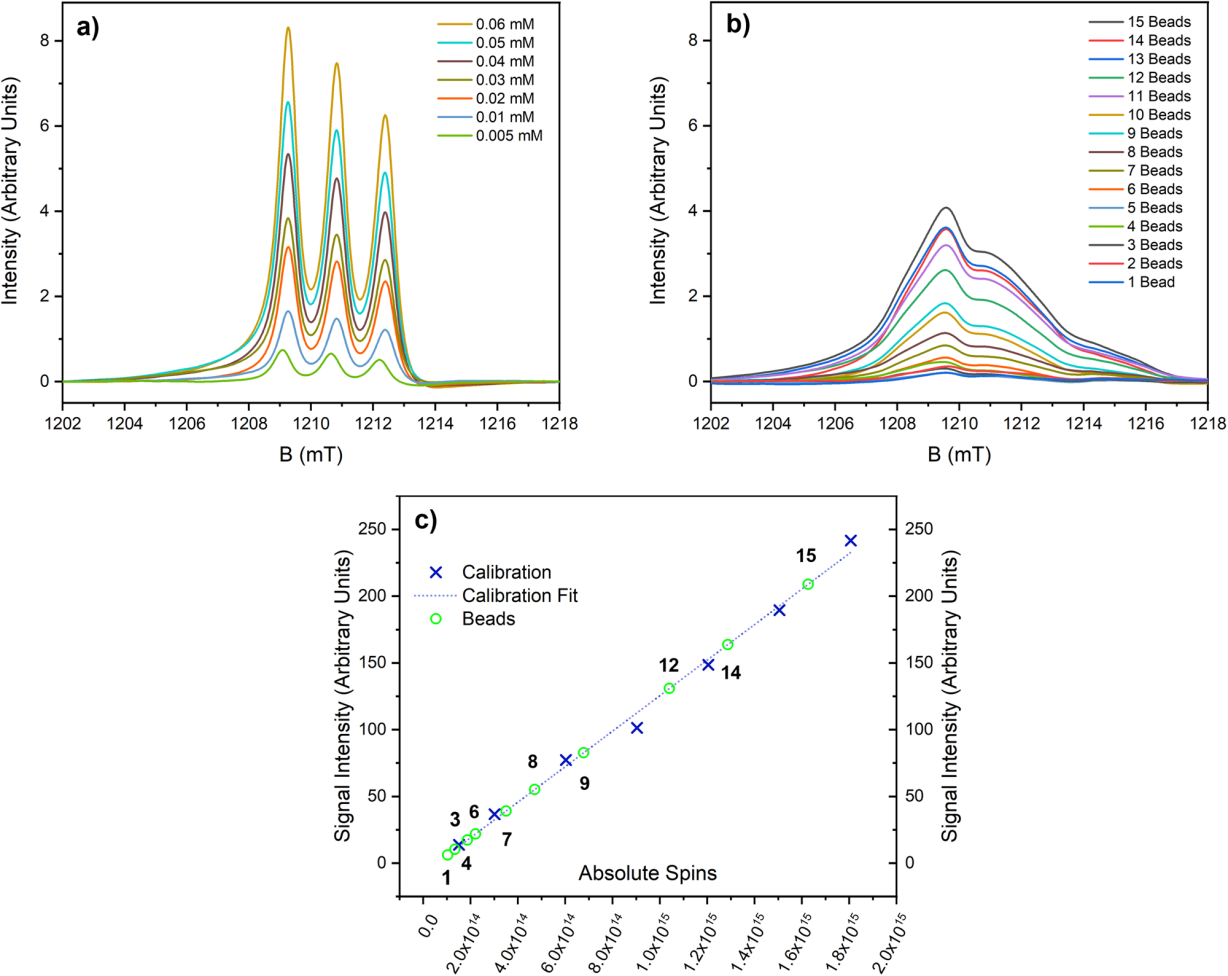
Spin counting on decorated beads. All spectra recorded at Q-band at room temperature. **a** First integral of Q-band EPR calibration solutions of **4** in toluene. **b** First integral of Q-band EPR spectra varying number of beads of **7**. **c** Spin counting on different numbers of beads **7**, calibrated against a solution of [2]rotaxane **4**. Black figures indicate the number of beads. Measurements for 2, 5, 10, 11 and 13 beads were omitted due to difficulties in obtaining accurate baseline corrections.

Spin counting measurements were then performed on beads of **7** (Fig. 4b), using increasing numbers of individual beads from one to fifteen (see SI, section 5 for full details). The signal intensities for the different numbers of beads are mapped onto the calibration curve in Fig. 4c.

The number of nitroxide spins per bead was found to be of the order 10^14^ (Table 2). Although the signal intensity increases with number of beads, it does not do so linearly. The detection limit of EPR at these frequencies, temperatures and microwave powers is ca. 10^12^ spins/mT line-width^22^. Hence, at the lower end of our concentration scale the accuracy of the spin counting experiment is very limited. Moreover, (i) with a single (or a few) beads, positioning of the sample in the EPR tube becomes more crucial, and (ii) there is a distribution of bead sizes with bead diameters between 80 and 150 μm. Hence, we consider the average number of nitroxide spins per bead from the larger numbers of beads to be the most meaningful. The conclusion is that there are ~10^14^ spins per bead, which is within an order of magnitude of the lowest estimate of the number of rotaxanes that could be bound (ca. 2 × 10^13^), calculated from the number of solvent-accessible surface sites (See SI, section 2).

**Table 2:** Spin counting of the TEMPO radical signal in **7** measured at 34 GHz and room temperature.

No. of beads	Total no. of spins/10^14^	No. of spins per bead/10^14^
6	2.2	0.4
7	3.5	0.5
8	4.7	0.6
9	6.8	0.8
12	10.4	0.9
14	12.9	0.9
15	16.3	1.1

In addition, 10 µm diameter beads **6** were decorated with **4** giving **8**, and these beads were also studied (Fig. S20). The mass of the beads measured (1.35 mg) equates to ~7.1 × 10^5^ beads. The signal intensity observed was compared with our calibration and this leads to 7.2 × 10^10^ spins/bead, which is within an order of magnitude from the estimate of the number of rotaxanes that could be bound (ca. 8 × 10^10^), calculated from the number of solvent-accessible surface sites (see SI, section 2). The nitroxide resonance in **4** and **8** is slightly broader than in **7**.

### Magnetic measurements

**Fig. 5:**
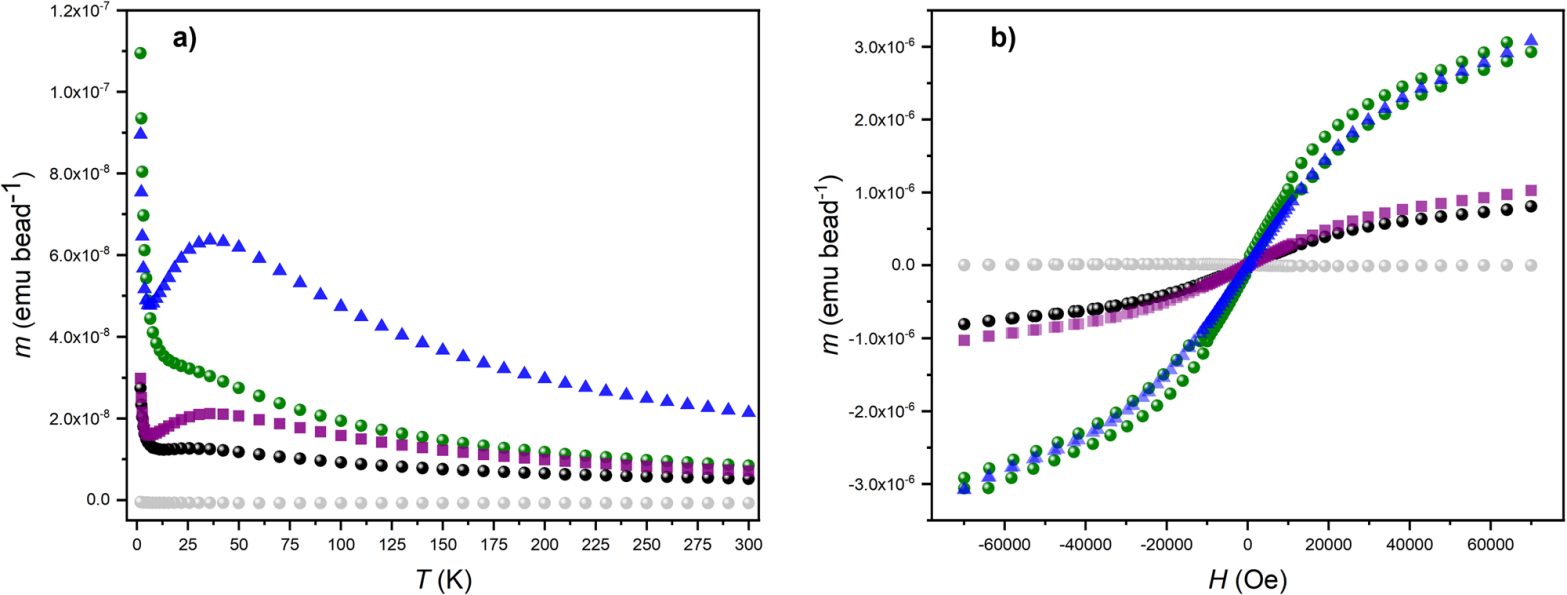
Magnetic properties of decorated beads. **a** Variable temperature moment (*m*) at 1000 Oe for beads scaled to one bead: **5** (grey), **7** (black), **11** (green), **12** calculated with 1 × 10^14^ rotaxanes (purple squares), **12** calculated with 3 × 10^14^ rotaxanes (blue triangles). **b** Variable field moment (*m*) at 2 K for beads scaled to one bead: **5** (grey), **7** (black), **11** (green), **12** calculated with 1 × 10^14^ rotaxanes (purple squares), **12** calculated with 3 × 10^14^ rotaxanes (blue triangles).

Measuring the magnetic properties of samples containing polystyrene beads presents significant challenges, particularly as there is considerable static between the beads within samples. Nonetheless we have attempted to measure the magnetic properties of three samples of beads: an azide-decorated bead **5**, **7** and a bead decorated with crystallographically characterised (see Table S1) [2]rotaxane **10** [{(HC≡CCH_2_CH_2_C(O)O-Ph-CH_2_)_2_NH_2_}][Cr_7_NiF_8_(O_2_C^t^Bu)_16_], forming **11** that does not contain the nitroxide radical on the thread (See Methods section for preparative details). The results are shown in Fig. 5 plotted per bead. The values per bead are based on counting ~830 beads per mg in a sample of **11**.

At room temperature the susceptibility of **7** and **11** are similar, especially given the limitations of the experiment, and higher than that of the beads alone. As the temperature is lowered the behaviour diverges (Fig. 5a). Both show a maximum at around 25 K in the moment, *m*, (equivalent to susceptibility, *χ*), which is found in pure samples of the {Cr_7_Ni} rings^10^. This indicates that the predominant paramagnetic species present are intact {Cr_7_Ni} rings. This led us to compare **7** and **11** with the magnetic behaviour of [Pr_2_NH_2_][Cr_7_NiF_8_(O_2_C^t^Bu)_16_] **12**. In Fig. 5a, b we include the molar value for moment for **12**, scaled by both 10^14^/*N*_A_ and 3 × 10^14^/*N*_A_ giving the value for 10^14^ and 3 × 10^14^ molecules.

At the highest temperatures the magnetic susceptibilities of both **7** and **11** match the scaled value for 10^14^ molecules of **12** well. At low *T* both the temperature and field dependence of the moment for **7** match that for 10^14^ molecules of **12**; this is unexpected as **7** contains an additional *s* = ½ centre per rotaxane due to the radical. When comparing with **11**, the moment in both variable *T* and *H* fits with 3 × 10^14^ molecules of **12**. The susceptibility of **11** diverges after the maximum at 25 K.

We stress there must be considerable errors in these preliminary experiments and it is important we do not over interpret. However, both the EPR and magnetic measurements indicate around 10^14^ rotaxanes per bead; this is close to the lowest estimate of 2 × 10^13^ we calculate based on solvent-accessible sites (see section S2 and Fig. S11). Given the limitations of the experiments the agreement is excellent. The difference seen between **7** and **11** may indicate some weak interactions are present in the beads but the evidence is inconclusive.

## Conclusions

Highly paramagnetic microparticles have been prepared using a very different route to traditional paramagnetic nanoparticles. We have shown we can attach around 10^14^ rotaxanes on beads of average diameter 115 μm, measuring the number of rotaxanes attached by both EPR spectroscopy and magnetometry; the number matches well with our estimate that at least 2 × 10^13^ sites are available on a 115 μm bead. This also demonstrates that CuAAC click chemistry could be generally useful for making highly unusual nanostructures containing hybrid inorganic–organic molecules and supramolecules. In the future we will examine whether such nanoparticles could be used in applications such as DNP^6^.

## Methods

### Materials

All chemicals were of reagent grade quality, and they were purchased from commercial sources and used as received. The azide functionalized polystyrene beads were prepared by literature procedures^16,17^ (see SI section 1.2). Full characterisation details of all compounds are given in the section S1 and S3 of the Supplemental Information.

### Physical techniques

^1^H NMR spectra were recorded on a Bruker AVIII HD 400 MHz at 298 K. Chemical shifts are reported in parts per million (ppm) from high to low frequency using the residual solvent peak as the internal reference (DMSO-*d*_6_ = 2.50 ppm). All ^1^H resonances are reported to the nearest 0.01 ppm. Infrared spectra were recorded neat on a Bruker FT-IR Alpha II Platinum ATR spectrometer. Fully characterised compounds were chromatographically homogeneous. Flash column chromatography was carried out using Silica 60 Å (particle size 40–63 μm, Sigma Aldrich, UK) as the stationary phase. Analytical TLC was performed on precoated silica gel plates (0.25 mm thick, 60 F254, Merck, Germany) and visualised using both short and longwave ultraviolet light in combination with standard laboratory stains (acidic potassium permanganate, acidic ammonium molybdate and ninhydrin). Low-resolution ESI mass spectrometry and high-resolution mass spectrometry was carried out by staff at the Mass Spectrometry Service, Department of Chemistry, The University of Manchester. Elemental analysis was carried out by the Analytical Service of the University of Manchester; metals analysis by Thermo iCap 6300 inductively coupled plasma optical emission spectroscopy (ICP-OES).

**Fig. 6:**
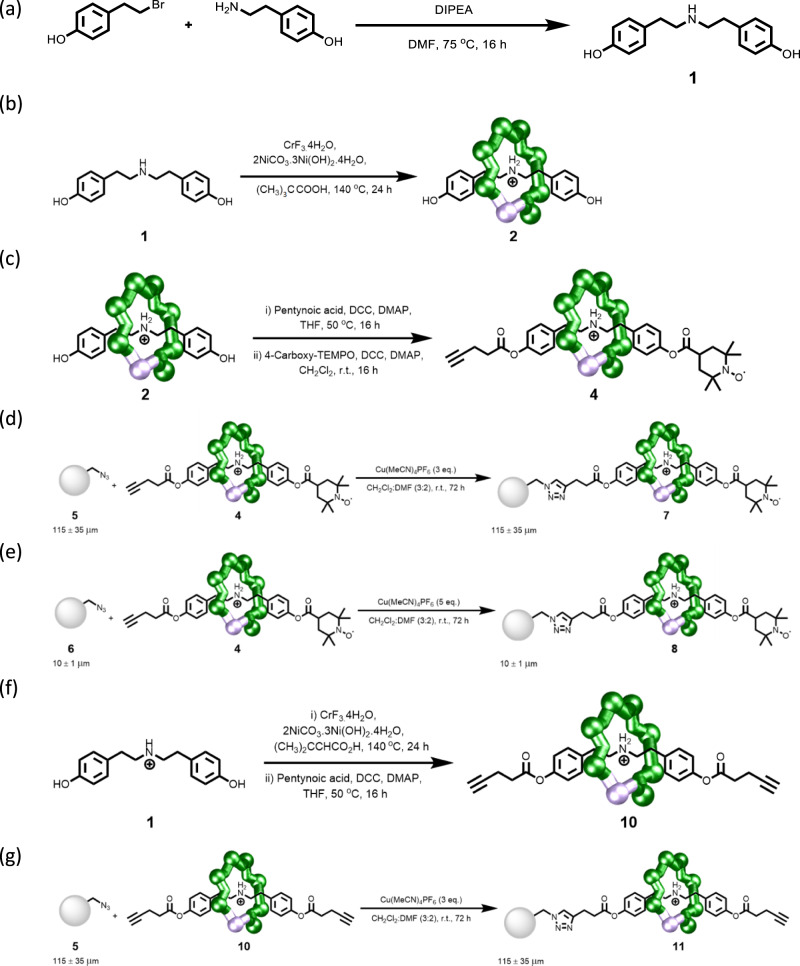
Synthetic reactions. **a** Synthesis of thread **1**. **b** Synthesis of **2**. **c** Synthesis of **4**. **d** Synthesis of **7**. **e** Synthesis of **8**. **f** Synthesis of **10**. **g** Synthesis of **11**.

### Synthesis of thread 1 (see Fig. 6a)

To a solution of 2-(4-hydroxyphenyl)−1-bromoethane (2.0 g, 10 mmol) in anhydrous DMF (45 mL) was added 2-(4-hydroxyphenyl)ethylamine (6.0 g, 44 mmol, 4.4 eq.) and *N*-ethyldiisopropylamine (5.2 mL, 30 mmol, 3 eq.). The reaction mixture was heated at 75 °C for 16 h. When the reaction was complete, the mixture was cooled to room temperature, diluted with EtOAc and washed with brine. The organic phase was dried (MgSO_4_), filtered, and the solvent removed under reduced pressure. The solid residue was dissolved in hot CH_3_OH (100 mL) and precipitated by addition of water (20 mL). The product was filtered and washed repeatedly with CHCl_3_ to yield the title compound as brown solid (1.8 g, 7.0 mmol, 70%).

### Synthesis of rotaxane 2 (see Fig. 6b)

To pivalic acid (45.0 g, 440 mmol) at 100 °C in a Teflon™ flask was added **1** (2.5 g, 9.7 mmol). The reaction mixture was stirred at 100 °C for 10 min then [CrF_3_·4H_2_O] (6.0 g, 33 mmol) and [2NiCO_3_·3Ni(OH)_2_·4H_2_O] (1.0 g, 1.7 mmol) were added. The reaction mixture was stirred at 140 °C for 24 h. The reaction mixture was cooled to room temperature and the crude product was extracted with CHCl_3_. The solvent was removed under reduced pressure and the crude residue was purified by column chromatography (SiO_2_, CHCl_3_:EtOAc, 90:10) to yield rotaxane **2** (3.5 g, 1.4 mmol, 15%).

### Synthesis of rotaxane 4 (see Fig. 6c)

To prepare molecule **4**, a two-step esterification was performed. Firstly, to a solution of rotaxane **2** (3.0 g, 1.2 mmol), in THF (100 mL) was added pentynoic acid (0.12 g, 1.2 mmol), DCC (0.90 g, 4.3 mmol) and DMAP (0.75 g, 6.1 mmol). The reaction mixture was stirred at 50 °C for 16 h. The solvent was removed under reduced pressure and the crude residue was purified by column chromatography (SiO_2_, CHCl_3_:EtOAc, 95:5) to yield mono-esterified rotaxane **3** (0.75 g, 0.30 mmol, 25%).

To a solution of rotaxane **3** (0.75 g, 0.30 mmol) in CH_2_Cl_2_ (50 mL) was added 4-carboxy-TEMPO (0.18 g, 0.90 mmol), DCC (0.55 g, 2.6 mmol) and DMAP (0.55 g, 4.5 mmol). The reaction mixture was stirred at room temperature for 16 h. The solvent was removed under reduced pressure and the crude residue was purified by column chromatography (SiO_2_, CHCl_3_:CH_3_OH, 95:5) to yield rotaxane **4** (0.65 g, 0.24 mmol, 80%).

### Synthesis of bead-rotaxane 7 (see Fig. 6d)

Azide polystyrene beads **5** (3.0 mg, 1.3 mmol g^−1^, 3.9 μmol) were added to [(C_31_H_40_N_2_O_5_)Cr_7_NiF_8_(C_5_H_9_O_2_)_16_] **4** (30 mg, 11 μmol, 3 eq.) and Cu(MeCN)_4_PF_6_ (4.0 mg, 11 μmol, 3 eq.) under argon. A CH_2_Cl_2_:DMF mixture (3:2, 5 mL, degassed with argon) was added and the reaction was stirred for 72 h at room temperature. The reaction mixture was then filtered and washed with CH_3_CN (3 × 10 mL), CH_3_OH (3 × 10 mL), and CH_2_Cl_2_ (3 × 10 mL) to afford [2]rotaxane–polystyrene beads **7** (13.0 mg, 3.7 μmol).

### Synthesis of bead-rotaxane 8 (see Fig. 6e)

Azide polystyrene beads **6** (2.5 mg, 1.0 mmol g^−1^, 2.5 μmol) were added to [(C_31_H_40_N_2_O_5_)Cr_7_NiF_8_(C_5_H_9_O_2_)_16_] **4** (30 mg, 11 μmol, 4 eq.) and Cu(MeCN)_4_PF_6_ (5.0 mg, 13 μmol, 5 eq.) under argon. A CH_2_Cl_2_:DMF mixture (3:2, 5 mL, degassed with argon) was added and the reaction was stirred for 72 h at room temperature. The reaction mixture was then filtered and washed with CH_3_CN (3 × 10 mL), CH_3_OH (3 × 10 mL), and CH_2_Cl_2_ (3 × 10 mL) to afford [2]rotaxane–polystyrene beads **8** (8.3 mg, 2.4 μmol).

### Synthesis of rotaxane 10 (see Fig. 6f)

To 3,3-dimethylacrylic acid (15.0 g, 150 mmol) in a Teflon™ flask was added **1** (1.5 g, 5.8 mmol) and 5.0 mL of 1,2-dichlorobenzene and heated to 140 °C for 10 min. To this solution [CrF_3_·4H_2_O] (3.0 g, 16 mmol) and [2NiCO_3_·3Ni(OH)_2_·4H_2_O] (0.75 g, 1.2 mmol) were added and stirred at 140 °C for 24 h. The reaction mixture was cooled to room temperature, transferred in to a 500 mL flask and 300 mL of hexane was added. The crude product was extracted with diethyl ether and purified by column chromatography (Silica, toluene/EtOAc) to yield rotaxane **9** (2.5 g). Single-crystal X-ray quality crystals were grown from acetone by slow evaporation method.

To prepare rotaxane **10**, to a solution of rotaxane **9** (2.0 g, 0.8 mmol), in THF (100 mL) was added pentynoic acid (0.2 g, 2.0 mmol), DCC (0.3 g, 1.4 mmol) and DMAP (0.25 g, 2.0 mmol). The reaction mixture was stirred at 50 °C for 16 h. The solvent was removed under reduced pressure and the crude residue was purified by column chromatography (SiO_2_, CHCl_3_:EtOAc, 95:5) to yield **10**. Single-crystal X-ray quality crystals were grown from acetone by slow evaporation method.

### Synthesis of bead-rotaxane 11 (see Fig. 6g)

Azide polystyrene beads **5** (7.0 mg, 1.3 mmol g^−1^, 9.0 μmol) were added to [(C_26_H_28_NO_4_)Cr_7_NiF_8_(C_5_H_7_O_2_)_16_] **10** (72 mg, 27 μmol, 3 eq.) and Cu(MeCN)_4_PF_6_ (10 mg, 27 μmol, 3 eq.) under argon. A CH_2_Cl_2_:DMF mixture (3:2, 5 mL, degassed with argon) was added and the reaction was stirred for 72 h at room temperature. The reaction mixture was then filtered and washed with CH_3_CN (3 × 10 mL), CH_3_OH (3 × 10 mL), and CH_2_Cl_2_ (3 × 10 mL) to afford [2]rotaxane–polystyrene beads **11** (28 mg, 8.8 μmol).

### Single-crystal X-ray diffraction

X-Ray data for compounds **2**, **4** and **9** were collected at a temperature of 100 K using a dual wavelengh Rigaku FR-X with Cu-Kα radiation equipped with a HypixHE6000 detector and an Oxford Cryosystems nitrogen flow gas system. X-ray data for compound **10** was collected at a temperature of 100 K using synchrotron radiation at beamline I19 in Diamond Light Source equipped with a Pilatus 2 M detector and an Oxford Cryosystems nitrogen flow gas system. Data were measured using GDA and CrysAlisPro suite of programmes.

X-Ray data were processed and reduced using CrysAlisPro suite of programmes. Absorption correction was performed using empirical methods (SCALE3 ABSPACK) based upon symmetry-equivalent reflections combined with measurements at different azimuthal angles^23^. The crystal structure was solved and refined against all *F*^2^ values using the SHELXL^24^ and Olex 2 suite of programmes^25^.

All atoms in crystal structures were refined anisotropically with the exception of the hydrogen atoms, that were placed in the calculated idealised positions for all crystal structures. The pivalate ligands, and threads in crystal structures were disordered and modelled over two positions, using structural same distance (SADI) and distance fix (DFIX) Shelxl restraints commands. The atomic displacement parameters (adp) of the ligands have been restrained using similar Ueq and rigid bond (SIMU) and Similar Ueq (SIMU) restraints.

### Spectroscopic measurements

Q-band (ca. 34 GHz) EPR spectra were recorded with a Bruker EMX580 spectrometer at the EPRSC UK National EPR Research Facility at The University of Manchester. The data were collected on polycrystalline powders and on solid polymeric beads at 280 K, and 5 K using liquid helium cooling. Spectral simulations were performed using the *EasySpin 4.5.5* simulation software^26^.

### Magnetic measurements

Magnetic properties were measured on a Quantum Design MPMS3. Susceptibility was measured from 300 to 2 K in a 1000 Oe magnetic field. Magnetisation was measured at 2 K to a maximum field of 7 T. Samples were mounted in gelatine capsules. The mass of samples measured were: **5**, 21.83 mg; **7**, 7.04 mg; **11**, 14.28 mg; **12**. 15.01 mg. No diamagnetic correction has been applied to data. For **7**, beads were counted and weighed and this leads to an estimate of 831 beads per mg, which we have used in calculations.

## Supplementary information

Supplemental Material

Description of Additional Supplementary Files

Supplementary Data 1

## Data Availability

Supplementary data are available in the online version of the paper. This includes Supplementary data 1, which includes the crystallographic data for this paper. These data have also been deposited as CCDC 2058841, 2058842, 2058844 and 2058846 numbers. These data can be obtained free of charge via www.ccdc.cam.ac.uk/conts/retrieving.html (or from the Cambridge Crystallographic Data Centre, 12 Union Road, Cambridge CB21EZ, UK; fax: (+44)1223-336-033; or deposit@ccdc.cam.ac.uk). Reprints and permissions information is available online at www.nature.com/reprints. Correspondence and requests for materials should be addressed to R.E.P.W.
