# Supplementary material for: Decorating polymer beads with 1014 inorganic-organic [2]rotaxanes as shown by spin counting
Source: Commun Chem. 2022 Jun 20;5:73. doi: 10.1038/s42004-022-00689-1 (PMC9814693; doi:10.1038/s42004-022-00689-1)
Supplement: Supplementary file 1 — Supplemental Material [file 42004_2022_689_MOESM1_ESM.pdf]

Supporting Information

Decorating polymer beads with 10^{14} inorganic-organic [2]rotaxanes as shown by spin counting

Deepak Asthana,[a,b] Dean Thomas,[a] Selena J. Lockyer,[a] Adam Brookfield,[a] Grigore A. Timco,[a] Iñigo J. Vitorica-Yrezabal,[a] George F. S. Whitehead,[a] Eric J. L. McInnes,[a] David Collison,[a] David A. Leigh[a] and Richard E. P. Winpenny[a]*

[a] *Department of Chemistry, The University of Manchester, Oxford Road, Manchester M13 9Pl, United Kingdom*

[b] *Department of Chemistry, Ashoka University, Sonipat, Haryana, India.*

**Correspondence: richard.winpenny@manchester.ac.uk*

Table of Contents **Page No.**

S1 Supplementary methods: characterization data

S1.1 Characterization data of **1-4** & **7-11** S1

S1.2 Synthesis of azide polystyrene beads **5** and **6** S8

S2 Supplementary discussion: **solvent-accessible surface sites calculation of polystyrene beads** S9

S3 Supplementary methods: crystallographic information S10-S12

S4 Supplementary methods: Infrared spectra S13-S19

S5 Supplementary methods: EPR measurement and spin counting experiments S20-S26

S6 Supplementary references S27

S1. Supplementary methods: characterization data

S1.1. Characterization data (1-4, 7-11)

$C_{16}H_{19}NO_2$ (1):

Elemental Analysis: Calculated (found) for $C_{16}H_{19}NO_2$: C, 74.68 (74.03); H, 7.44 (7.45); N, 5.44 (5.45).

^{1}H NMR (400 MHz, DMSO-d_6, 300 K): δ 9.15 (2H, Ph-O*H*), 6.95 (4H), 6.64 (4H), 2.67 (4H, Ph-CH_2), 2.55 (4H, Ph-CH_2CH_2), 1.43 (1H, N*H*).

$C_{96}H_{164}Cr_7F_8NNiO_{34}$ (2):

ESI-MS ($C_{96}H_{164}Cr_7F_8NNiO_{34}$, M.W.= 2450.9): 2473.6 $(M+Na)^+$

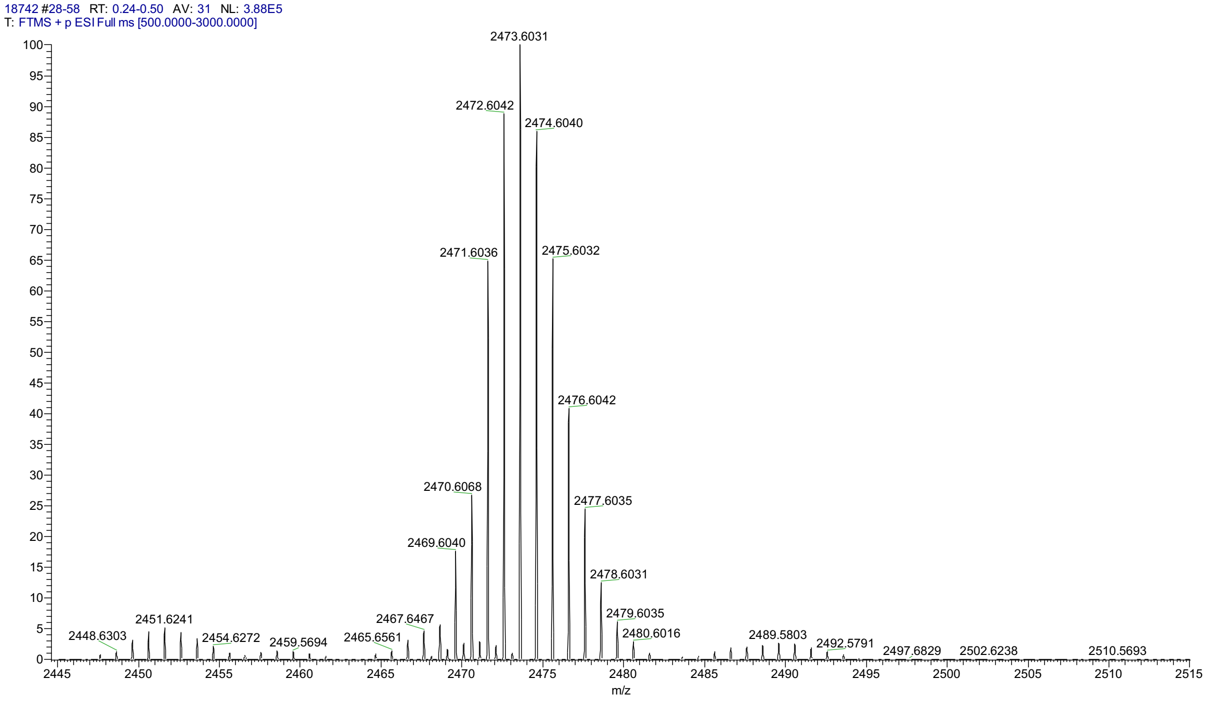

Figure S1. Experimental ESI-MS Spectrum of Rotaxane **2**:

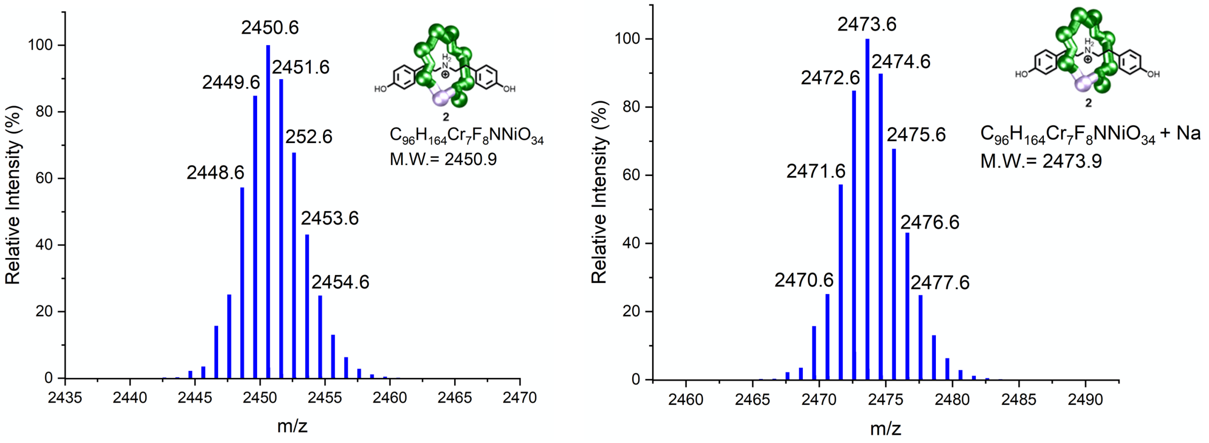

Figure S2. Calculated MS for Rotaxane **2** and Rotaxane **2 + Na**:

Elemental Analysis: Calculated (found) for $C_{96}H_{164}Cr_7F_8NNiO_{34}$: C, 47.04 (47.35); H, 6.74 (6.84); N, 0.57 (0.65); Cr, 14.85 (13.78); Ni, 2.39 (2.32).

$C_{101}H_{168}Cr_7F_8NNiO_{35}$ (3):

ESI-MS ($C_{101}H_{168}Cr_7F_8NNiO_{35}$, M.W.= 2531.1): 2553.6 $(M+Na)^+$

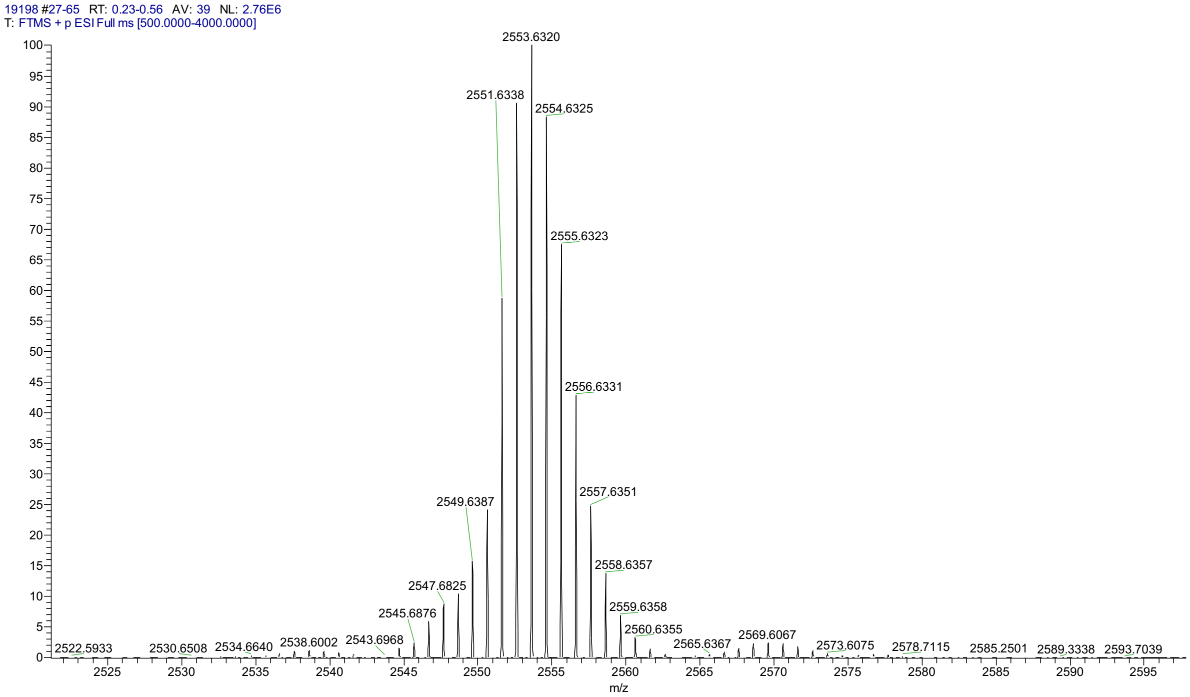

Figure S3. Experimental ESI-MS Spectrum of Rotaxane **3**:

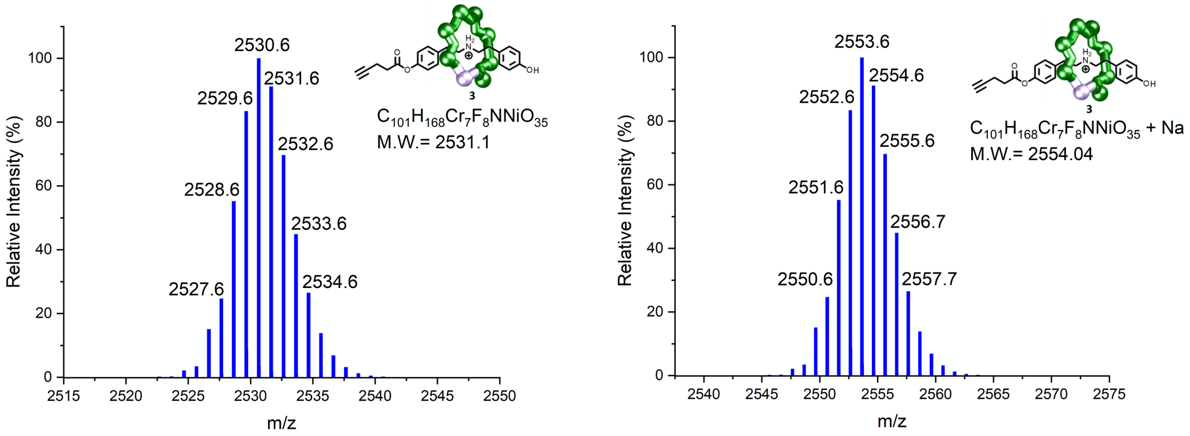

Figure S4. Calculated MS for Rotaxane **3** and Rotaxane **3** + **Na**:

Elemental Analysis: Calculated (Found) for $C_{101}H_{168}Cr_7F_8NNiO_{35}$: C, 47.93 (48.11); H, 6.69 (6.85); N, 0.55 (0.61); Cr, 14.38 (13.58); Ni, 2.32 (2.30).

$C_{111}H_{184}Cr_7F_8N_2NiO_{37}$ (4):

ESI-MS ($C_{111}H_{184}Cr_7F_8N_2NiO_{37}$, M.W.= 2713.3): 2713.7 $(M+H)^+$

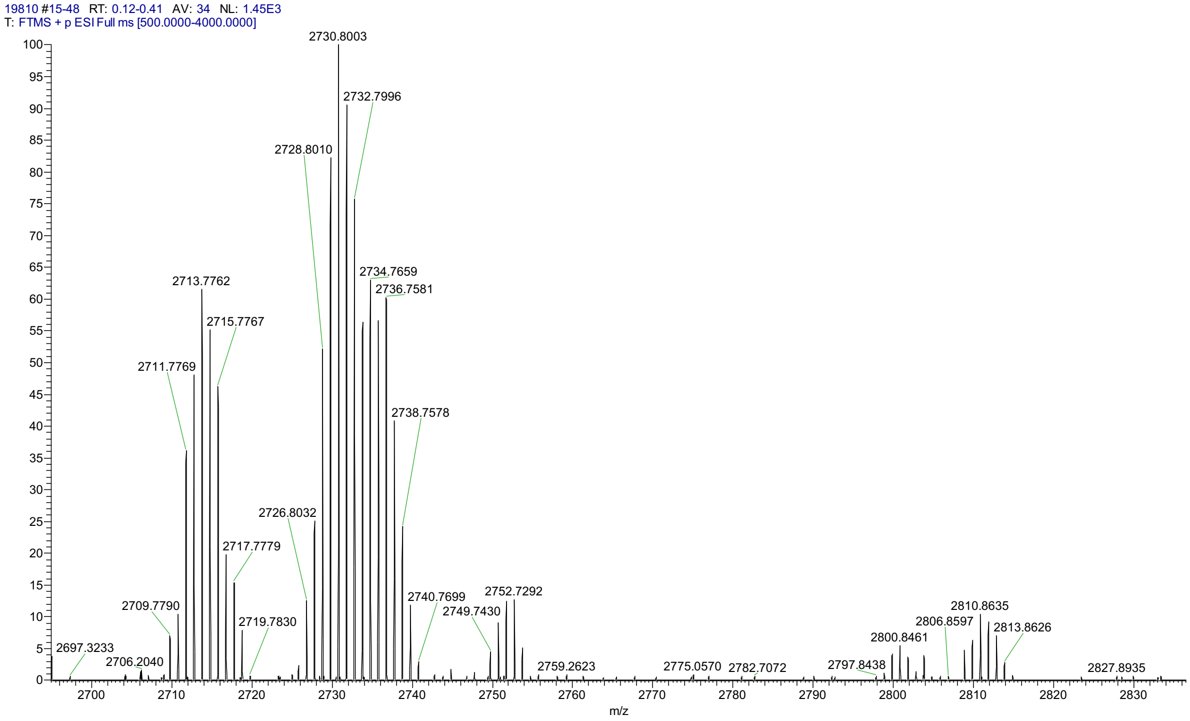

Figure S5. Experimental ESI-MS spectrum of Rotaxane **4**:

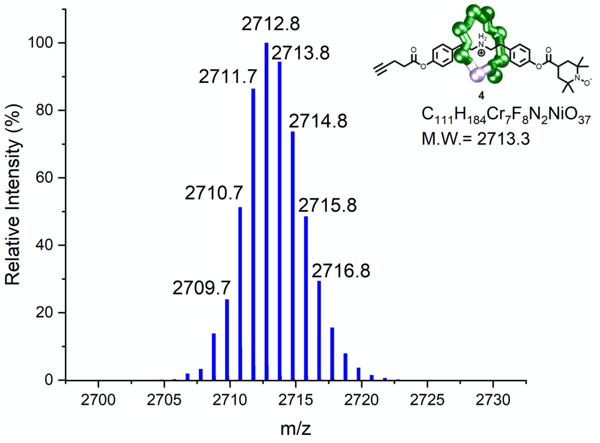

Figure S6. Calculated MS for Rotaxane **4**:

Elemental Analysis: Calculated (Found) for $C_{111}H_{184}Cr_7F_8N_2NiO_{37}$: C, 49.14 (50.11); H, 6.84 (6.94); N, 1.03 (1.39); Cr, 13.41 (12.37); Ni, 2.16 (2.11).

IR (Powder, ν_{max} / cm^{-1}): 2965, 2932, 2903, 2868, 1764, 1609, 1560, 1484, 1428, 1414, 1381, 1360, 1228, 1201, 1137, 1032, 901, 783, 616, 511.

[2]rotaxane–polystyrene beads (**7**):

IR (Powder, ν_{max} / cm^{-1}): 2967, 2928, 1762, 1607,1560, 1484, 1430, 1414, 1381, 1362, 1230, 1203, 1168, 1137, 1028, 905, 845, 783, 752, 697, 618 and 513.

[2]rotaxane–polystyrene beads (**8**):

IR (Powder, ν_{max} / cm^{-1}): 2963, 2930, 1762, 1609, 1560, 1484, 1428, 1412, 1381, 1360, 1228, 1201, 1168, 1131, 1030, 901, 783, 754, 699, 618, and 513.

$C_{96}H_{132}Cr_7F_8NNiO_{34}$ (**9**):

ESI-MS ($C_{96}H_{132}Cr_7F_8NNiO_{34}$, M.W.= 2418.7): 2441.3 $(M+Na)^+$

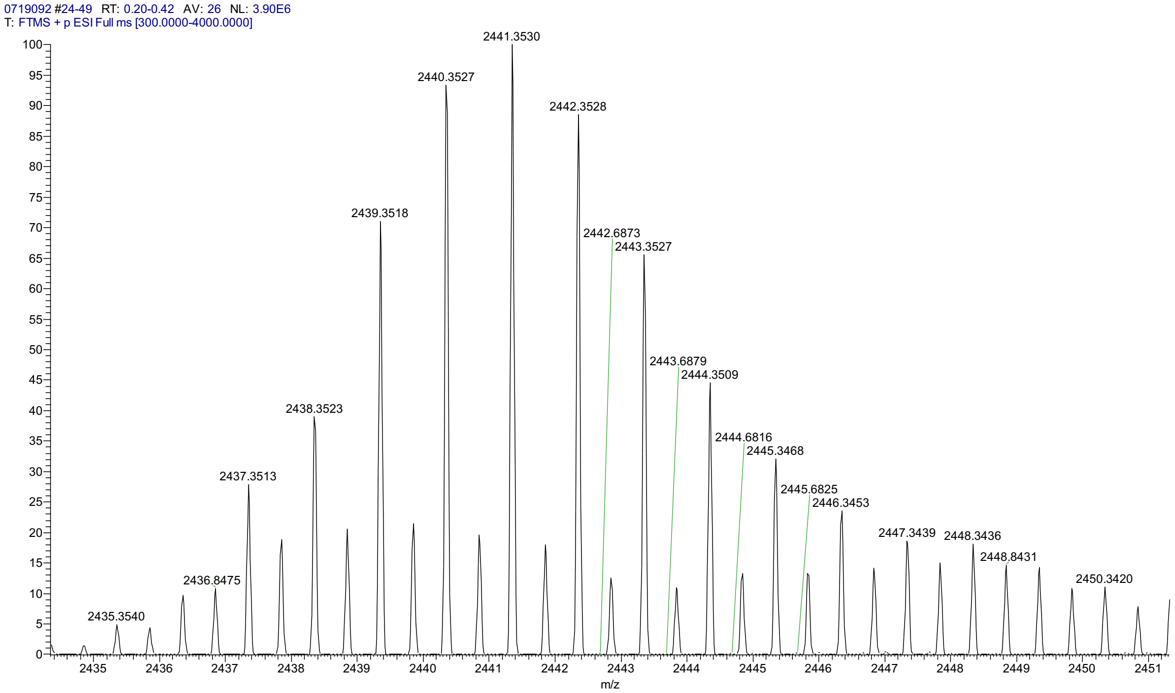

Figure S7. Experimental ESI-MS spectrum of Rotaxane **9**:

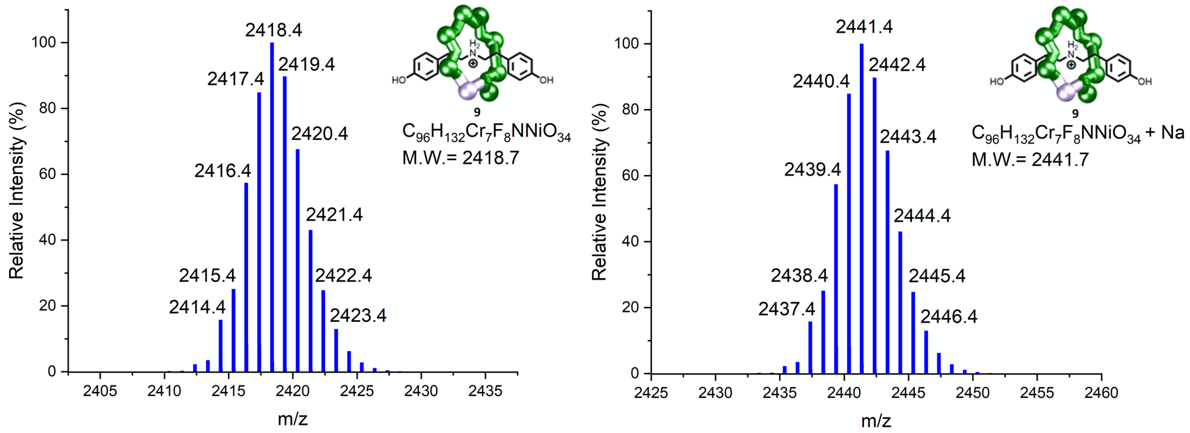

Figure S8. Calculated MS for Rotaxane **9** and Rotaxane **9 + Na**:

Elemental Analysis: Calculated (found) for $C_{96}H_{132}Cr_7F_8NNiO_{34}$: C, 47.67 (47.06); H, 5.50 (5.61); N, 0.58 (0.61); Cr, 15.05 (14.37); Ni, 2.43 (2.39).

$C_{106}H_{140}Cr_7F_8NNiO_{36}$ (10):

ESI-MS ($C_{106}H_{140}Cr_7F_8NNiO_{36}$, M.W.= 2578.9): 2601.3 $(M+Na)^+$

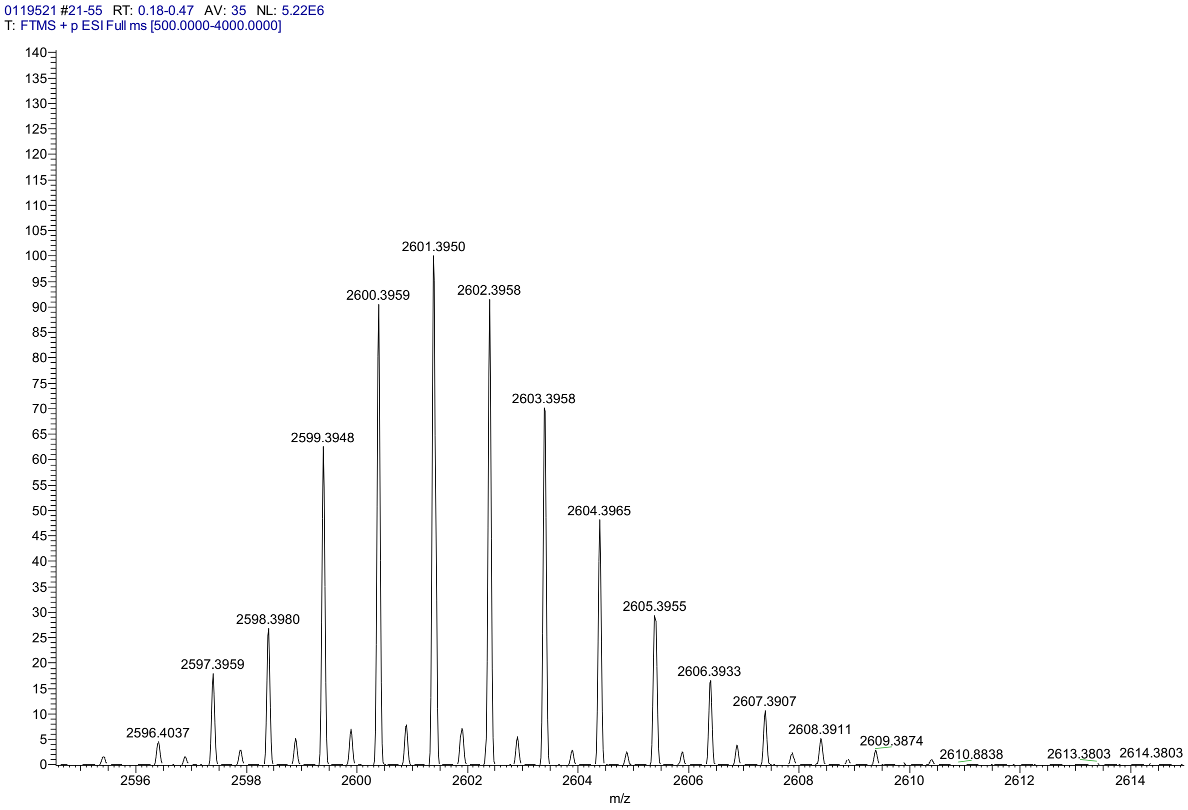

Figure S9. Experimental ESI-MS spectrum of Rotaxane **10**:

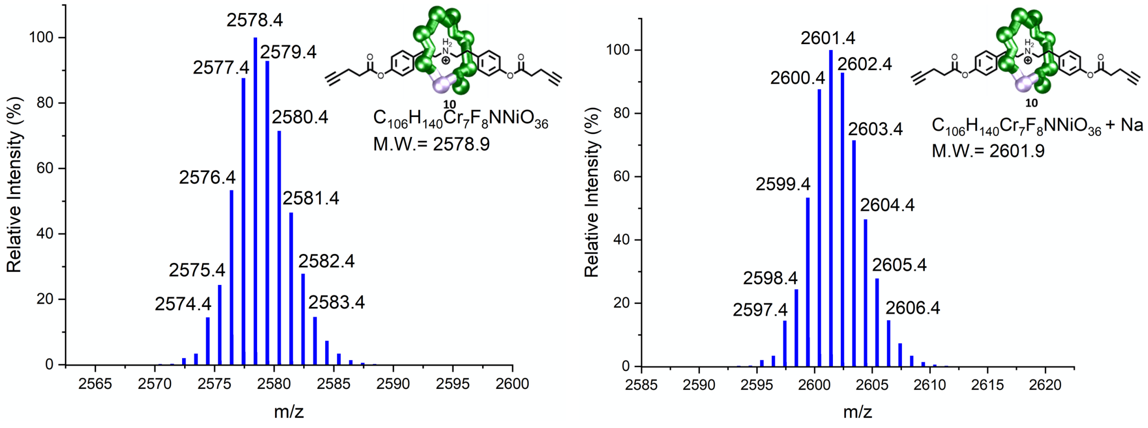

Figure S10. Calculated MS for Rotaxane **10** and Rotaxane **10 + Na**:

Elemental Analysis: Calculated (Found) for $C_{106}H_{140}Cr_7F_8NNiO_{36}$: C, 49.37 (49.34); H, 5.47 (5.44); N, 0.54 (0.60); Cr, 14.11 (13.85); Ni, 2.28 (2.27).

IR (Powder, v_{max} / cm^{-1}): 2963, 2930, 2906, 2868, 1766, 1609, 1560, 1535, 1510, 1484, 1428, 1414, 1381, 1360, 1228, 1201, 1168, 1137, 1032, 938, 900, 785, 616, 511, 490, 470.

[2]rotaxane–polystyrene beads (11):

IR (Powder, ν_{max} / cm^{-1}): 3025, 2959, 2928, 2864, 1760, 1702, 1607, 1560, 1484, 1453, 1430, 1414, 1381, 1360, 1228, 1203, 1168, 1133, 1049, 1028, 1004, 969, 941, 843, 783, 736, 699, 616, 511, 490, 472.

S1.2. Azide Bead Synthesis:

Synthesis of azide polystyrene beads (5):

Prepared in accordance with literature procedures.[1,2]

Merrifield resin beads (5.0 g, 1.3 mmol g^{-1}, 6.5 mmol) were added to NaN_3 (2.1 g, 32.5 mmol, 5 eq.) in DMSO (40 mL) and heated at 80 °C for 72 h under nitrogen. After being cooled to room temperature, the reaction mixture was filtered and washed with DMF (5 × 30 mL), CH_3OH (5 × 30 mL), CH_2Cl_2 (5 × 30 mL), and Et_2O (2 × 30 mL) to afford azide polystyrene beads **5** (5.0 g, 6.5 mmol).

IR (Powder, ν_{max} / cm^{-1}): 3083, 3058, 3025, 2920, 2850, 2094, 1601, 1492, 1451, 1341, 1249, 1203, 1181, 1154, 1115, 1067, 1028, 905, 841, 816, 752, 694 and 538.

Data in accordance with literature.[1,2]

Synthesis of azide polystyrene beads (6):

Adapted from literature procedures.[1,2]

Bromoethyl-polystyrene beads (250 mg, 1.0 mmol g^{-1}, 0.25 mmol) were added to NaN_3 (81 mg, 1.25 mmol) in DMSO (5 mL) and heated at 80 °C for 72 h under nitrogen. After being cooled to room temperature, the reaction mixture was filtered and washed with DMF (5 × 30 mL), CH_3OH (5 × 30 mL), CH_2Cl_2 (5 × 30 mL), and Et_2O (2 × 30 mL) to afford azide polystyrene beads **6** (240 mg, 0.24 mmol).

IR (Powder, ν_{max} / cm^{-1}): 3081, 3058, 3025, 2918, 2846, 2090, 1601, 1492, 1451, 1259, 1028, 907, 756, 697 and 540.

S2. Supplementary discussion: solvent-accessible surface sites calculation of polystyrene beads

The number of solvent-accessible surface sites of the initial polystyrene beads was estimated to determine the number of rotaxanes. The calculation leads to a lower bound of at least 1.9 x 10^{13} sites and 8.4 x 10^{10} sites per 115 µm and 10 µm polystyrene bead, respectively (Figure S1). The polystyrene beads are quantitatively converted to azide polystyrene beads[1,2] **5** and **6** before a CuAAC reaction with excess alkyne **4**. Therefore, an estimated lower bound for the number of rotaxanes per 115 µm and 10 µm polystyrene bead, in the solvent-accessible surface sites, is approximately 2 x 10^{13} and 8 x 10^{10}, respectively.

For 115 µm beads:

For 10 µm beads:

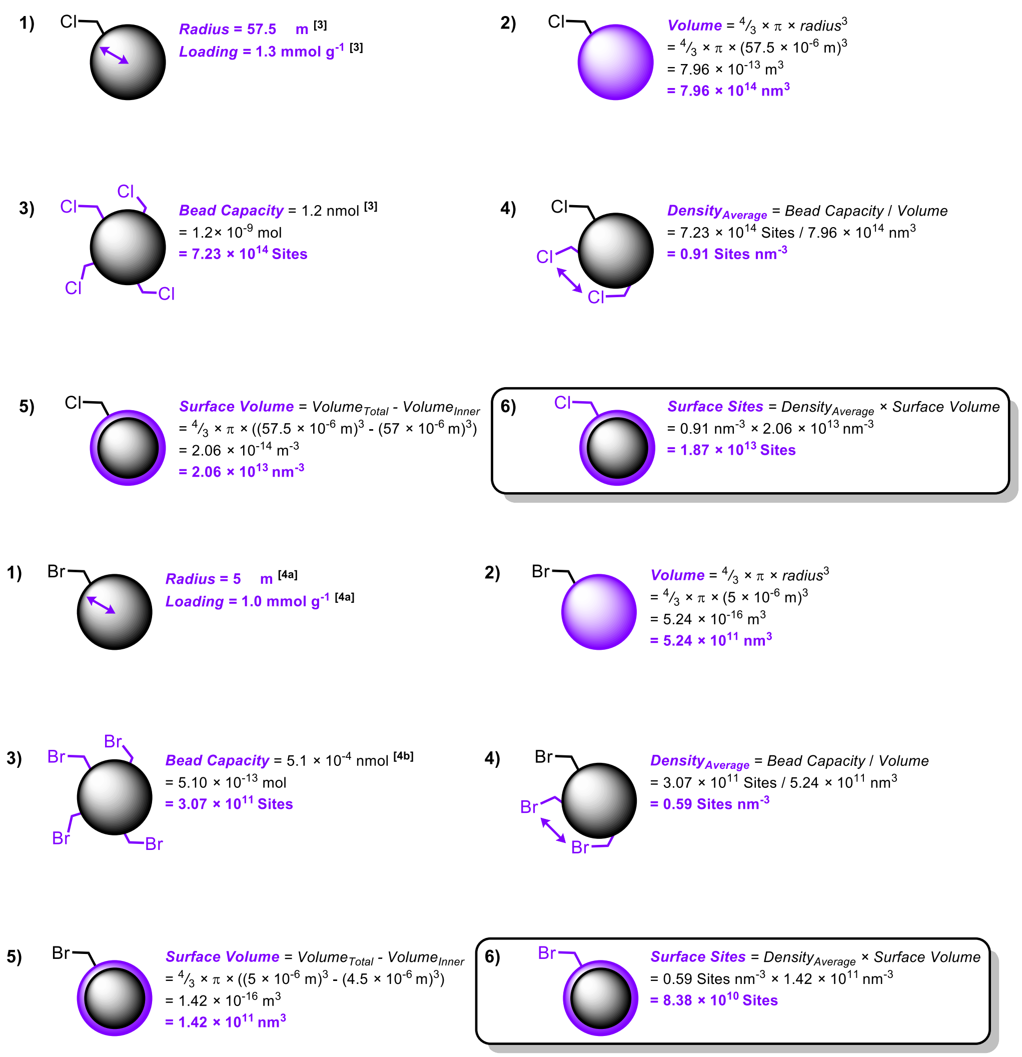

Figure S11. Solvent-Accessible Surface Sites Calculation. The assumptions for each step are as follows: 1) The manufacturer's specification describing the beads is accurate.[1-4] 2) The volume of the polymer bead can be approximated as a sphere. 3) The manufacturer's specification describing the capacity per bead is accurate and of the entire polymer.[1-4] 4) The density of sites is uniform across the polymer bead. 5a) The number of accessible surface sites is not limited to the surface of the bead as there is a degree of swelling and 'depth' in which molecules can react at sites further inside the sphere. A lower bound depth has been approximated to 0.5 µm. 5b) The surface volume can be determined from the difference of two spheres. 6) The density of polymer sites is uniform at the surface. 7) The subsequent azide formation and click reaction proceed quantitatively.[1,2]

S3. Supplementary methods: crystallographic Information

Data Collection. X-Ray data for compounds **2, 4** and **9** were collected at a temperature of 100 K using a dual wavelength Rigaku FR-X with Cu-Kα radiation equipped with a HypixHE6000 detector and an Oxford Cryosystems nitrogen flow gas system. X-ray data for compound **10** were collected at a temperature of 100 K using synchrotron radiation at beamline I19 in Diamond Light Source equipped with a Pilatus 2M detector and an Oxford Cryosystems nitrogen flow gas system. Data were measured using GDA and CrysAlisPro suite of programs.

Crystal structure determinations and refinements. X-Ray data were processed and reduced using CrysAlisPro suite of programs. Absorption correction was performed using empirical methods (SCALE3 ABSPACK) based upon symmetry-equivalent reflections combined with measurements at different azimuthal angles.[5] The crystal structure was solved and refined against all F^2 values using the SHELXL and Olex 2 suite of programmes.[6]

All atoms in crystal structures were refined anisotropically with the exception of the hydrogen atoms, that were placed in the calculated idealized positions for all crystal structures. The pivalate ligands, and threads in crystal structures were disordered and modelled over two positions, using structural same distance (SADI) and distance fix (DFIX) Shelxl restraints commands. The atomic displacement parameters (adp) of the ligands have been restrained using similar Ueq and rigid bond (SIMU) and Similar Ueq (SIMU) restraints.

CCDC 2058841, 2058842, 2058844 and 2058846 contains the supplementary crystallographic data for this paper. These data can be obtained free of charge *via* www.ccdc.cam.ac.uk/conts/retrieving.html (or from the Cambridge Crystallographic Data Centre, 12 Union Road, Cambridge CB21EZ, UK; fax: (+44)1223-336-033; or deposit@ccdc.cam.ac.uk).

Table S1. Crystallographic information for **2, 4**, **9** and **10**

Identification code	**2**	**4**
Empirical formula	$C_{106.8}H_{189.6}Cr_7F_8NNiO_{39.6}$	$C_{120}H_{202}Cr_7F_8N_2NiO_{40}$
Formula weight	2696.09	2887.53
Temperature/K	100.00(10)	100.00(10)
Crystal system	monoclinic	monoclinic
Space group	$P2_1/c$	C2/c
a/Å	25.82433(11)	58.8525(10)
b/Å	34.67902(19)	16.5562(3)
c/Å	81.9080(4)	31.1637(4)
α/°	90	90
β/°	90.8052(4)	92.731(2)
γ/°	90	90
Volume/$Å^3$	73346.5(6)	30330.6(8)
Z	20	8
$\rho_{calc}g/cm^3$	1.221	1.265
μ/mm^{-1}	4.902	4.781
F(000)	28444.0	12192.0
Crystal size/mm^3	0.28 × 0.24 × 0.18	0.55 × 0.43 × 0.33
Radiation (Å)	Cu Kα (λ = 1.54184)	Cu Kα (λ = 1.54184)
2Θ range for data collection/°	3.338 to 141.608	3.006 to 152.486
Index ranges	-29 ≤ h ≤ 31, -41 ≤ k ≤ 42, -80 ≤ l ≤ 100	-74 ≤ h ≤ 71, -7 ≤ k ≤ 20, -39 ≤ l ≤ 39
Reflections collected	416985	85389
Independent reflections	131133 [R_{int} = 0.0801, R_{sigma} = 0.0893]	30626 [R_{int} = 0.0454, R_{sigma} = 0.0593]
Data/restraints/parameters	131133/4224/8199	30626/532/1796
Goodness-of-fit on F^2	1.033	1.068
Final R indexes [I>=2σ (I)]	R_1 = 0.0787, wR_2 = 0.2024	R_1 = 0.0772, wR_2 = 0.2216
Final R indexes [all data]	R_1 = 0.1196, wR_2 = 0.2273	R_1 = 0.0949, wR_2 = 0.2371
Largest diff. peak/hole / e $Å^{-3}$	1.64/-1.07	1.21/-0.87

Identification code	**9**	**10**
Empirical formula	$C_{100}H_{142}Cr_7F_8N_3NiO_{36}$	$C_{106}H_{140}Cr_7F_8NNiO_{36}$
Formula weight	2536.87	2578.89
Temperature/K	100.00(10)	100.0(2)
Crystal system	monoclinic	monoclinic
Space group	C2/c	I2/a
a/Å	23.80098(18)	29.0196(6)
b/Å	20.26733(15)	16.6767(3)
c/Å	23.72358(16)	28.3605(6)
α/°	90	90
β/°	93.3793(6)	99.910(2)
γ/°	90	90
Volume/$Å^3$	11423.94(14)	13520.3(5)
Z	4	4
ρ_{calc}g/cm^3	1.475	1.267
μ/mm^{-1}	6.246	0.699
F(000)	5276.0	5356.0
Crystal size/mm^3	0.32 × 0.15 × 0.1	0.34 × 0.23 × 0.15
Radiation (Å)	Cu Kα (λ = 1.54184)	synchrotron (λ = 0.6889)
2Θ range for data collection/°	9.242 to 152.762	3.596 to 50.332
Index ranges	-30 ≤ h ≤ 28, -25 ≤ k ≤ 25, -26 ≤ l ≤ 29	-35 ≤ h ≤ 35, -20 ≤ k ≤ 20, -35 ≤ l ≤ 35
Reflections collected	35357	88457
Independent reflections	11509 [Rint = 0.0240, Rsigma = 0.0259]	13310 [R_{int} = 0.0431, R_{sigma} = 0.0206]
Data/restraints/parameters	11509/91/741	13310/4513/1417
Goodness-of-fit on F^2	1.032	1.674
Final R indexes [I>=2σ (I)]	R1 = 0.0432, wR2 = 0.1185	R_1 = 0.1144, wR_2 = 0.3507
Final R indexes [all data]	R1 = 0.0462, wR2 = 0.1206	R_1 = 0.1202, wR_2 = 0.3631
Largest diff. peak/hole / e $Å^{-3}$	0.71/-1.44	1.03/-0.59

S4. Supplementary data: IR Spectra

IR Spectrum of [($C_{31}H_{40}N_2O_5$)Cr_7NiF_8($C_5H_9O_2$)$_{16}$] (4):

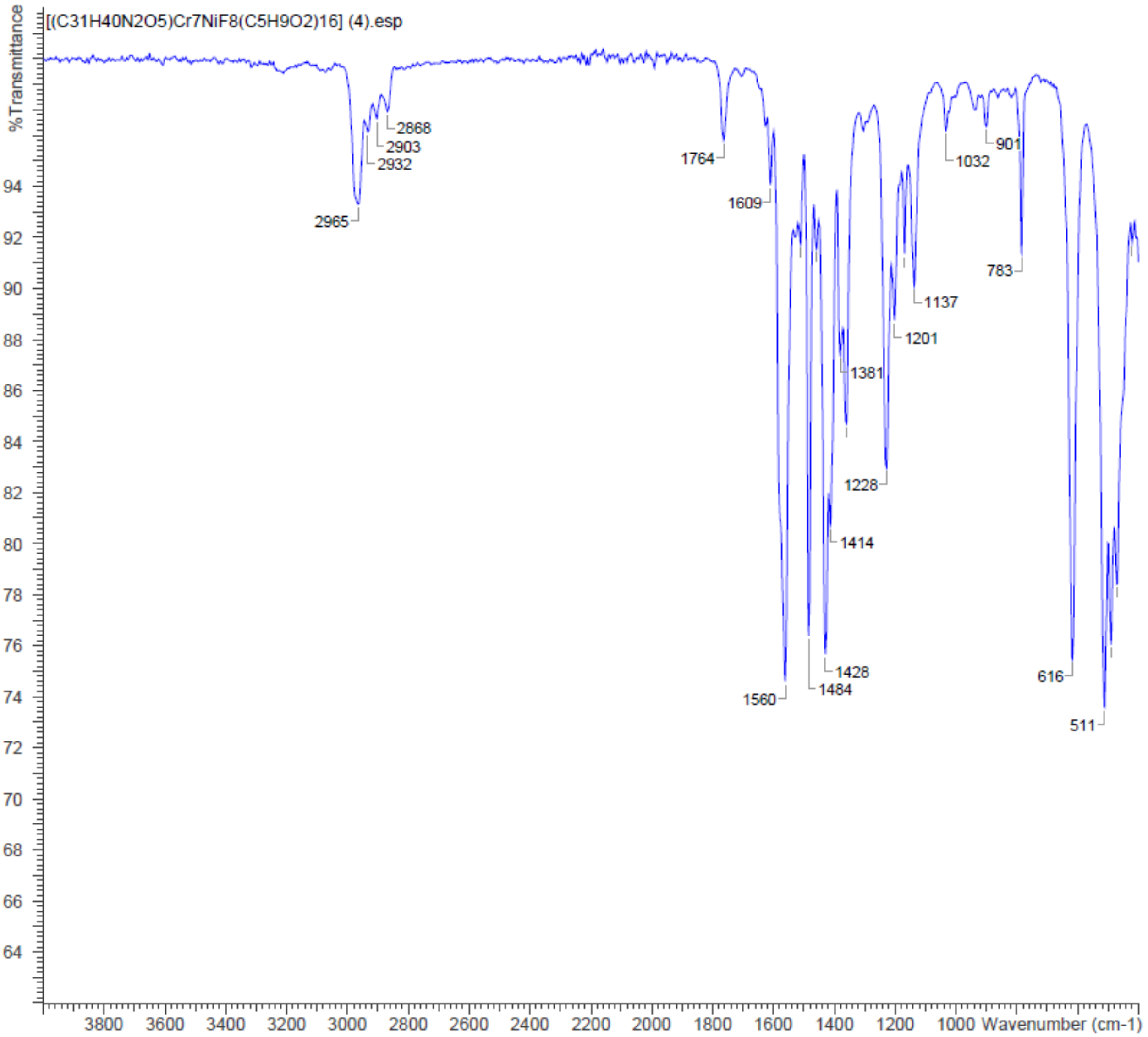

Figure S12 – Peak picked FT-IR spectrum of [($C_{31}H_{40}N_2O_5$)Cr_7NiF_8($C_5H_9O_2$)$_{16}$] **4**.

IR Spectrum of azide polystyrene beads (5):

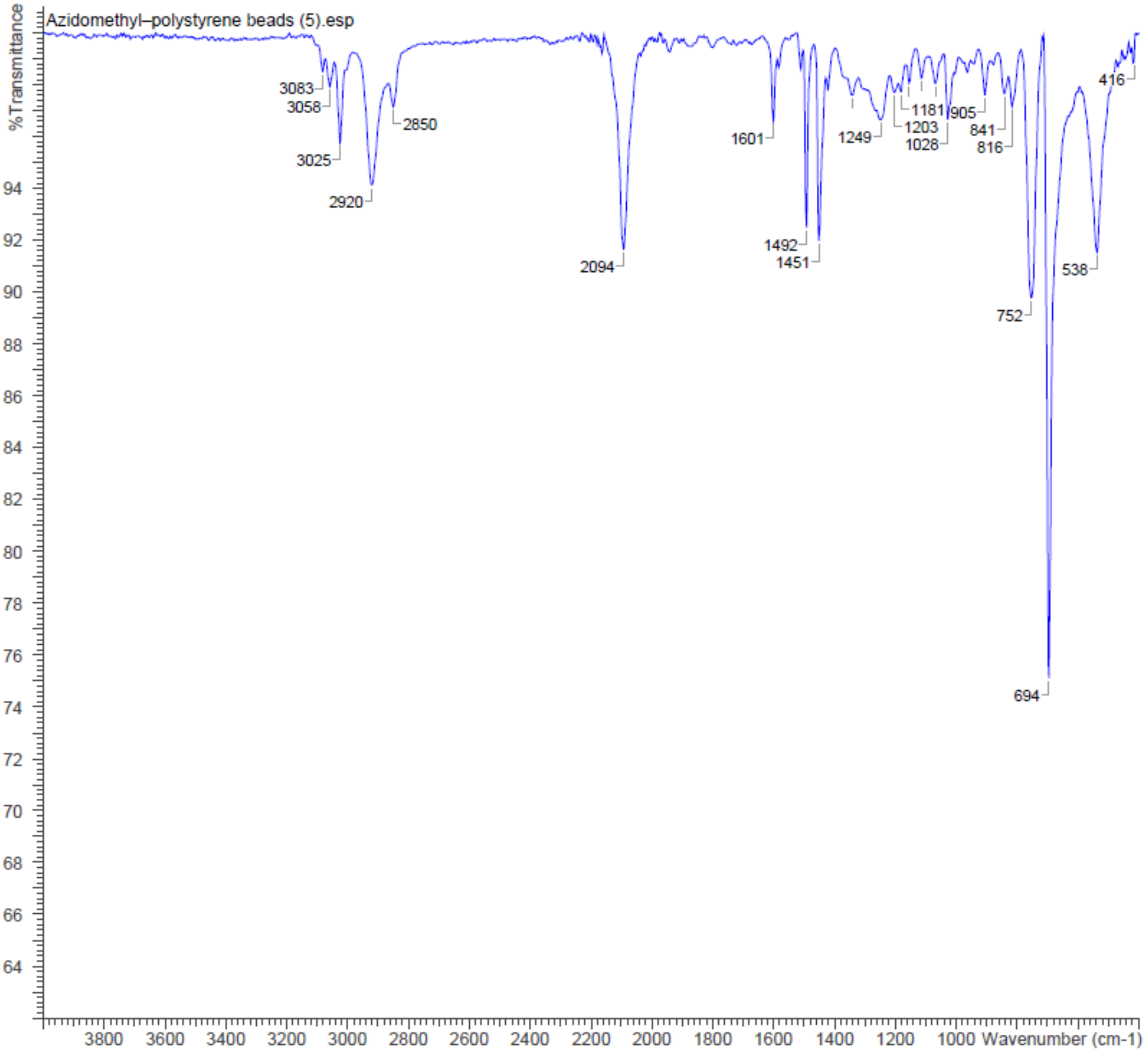

Figure S13 – Peak picked FT-IR spectrum of azide polystyrene beads **5**.

IR Spectrum of azide polystyrene beads (6):

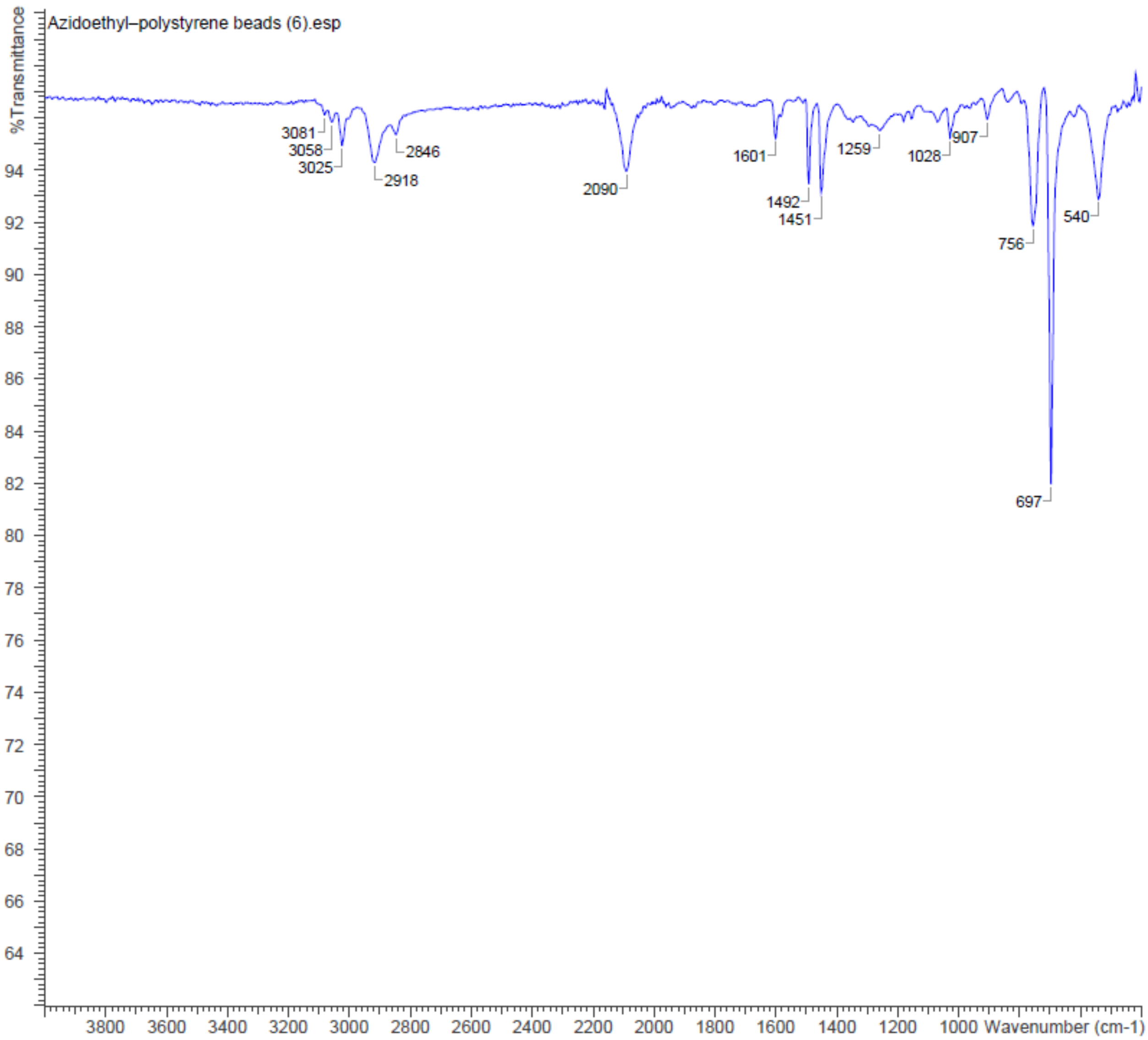

Figure S14 – Peak picked FT-IR spectrum of azide polystyrene beads **6**.

IR Spectrum of [2]rotaxane–polystyrene beads (7):

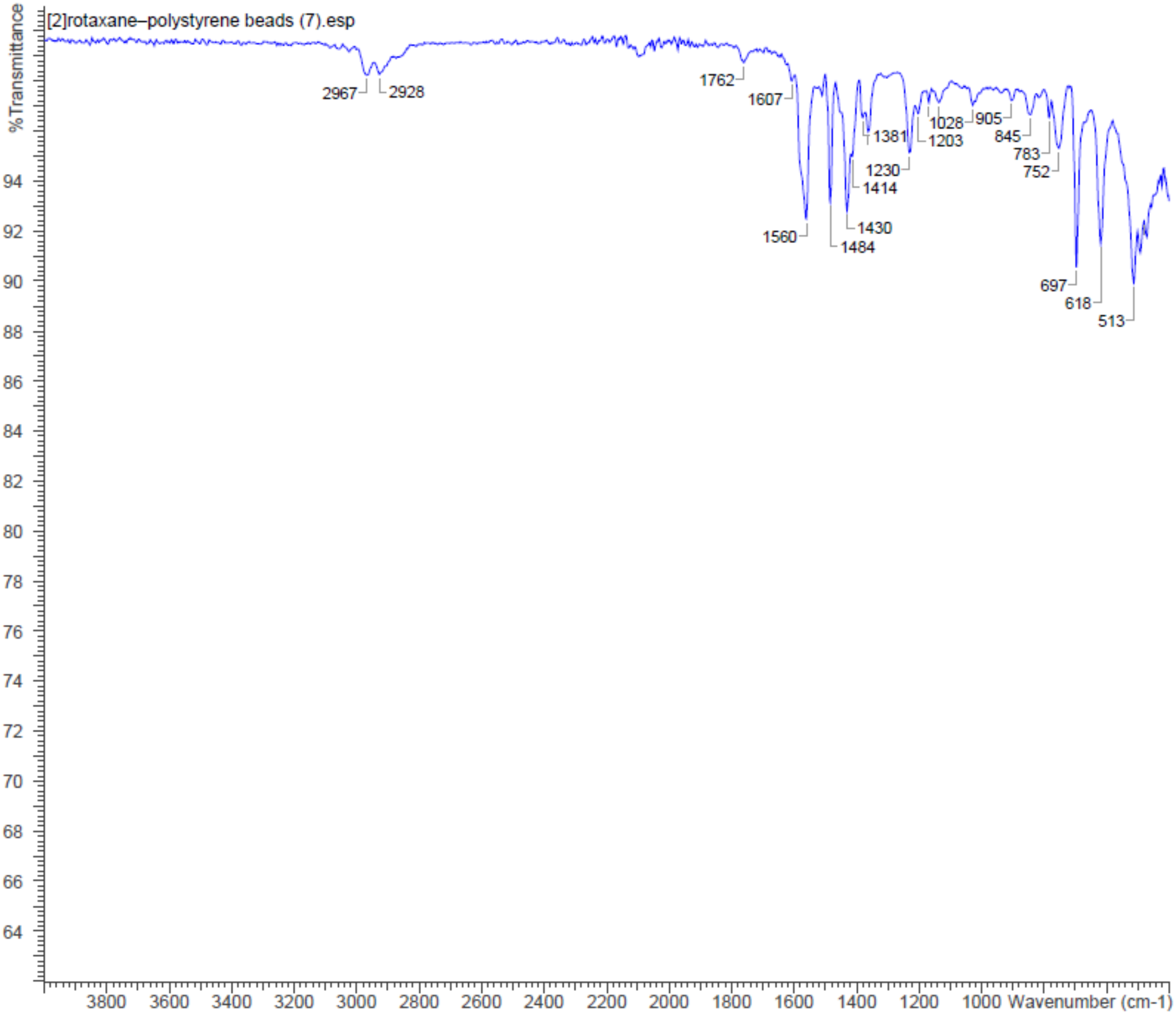

Figure S15 – Peak picked FT-IR spectrum of [2]rotaxane–polystyrene beads **7**.

IR Spectrum of [2]rotaxane–polystyrene beads (8):

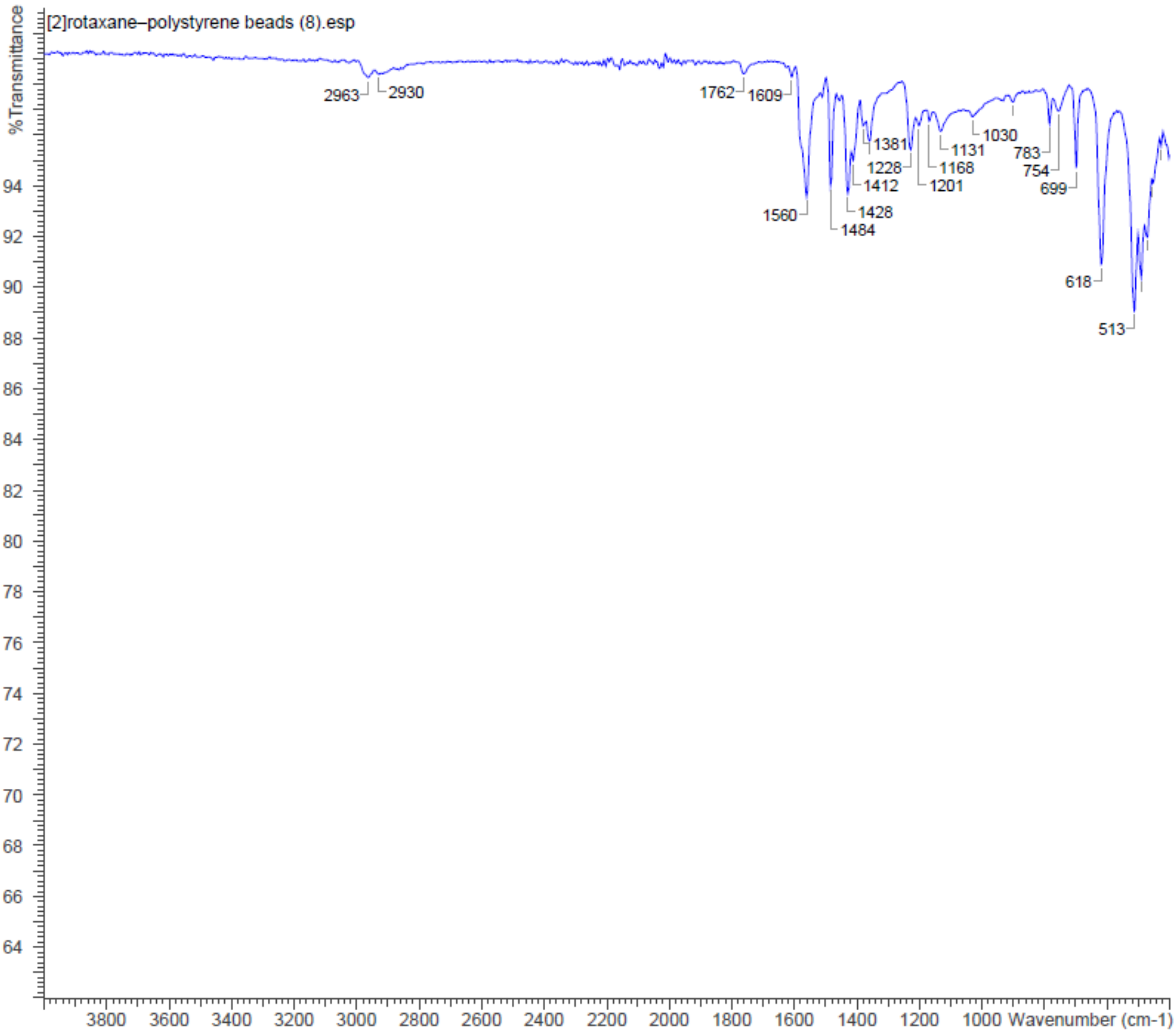

Figure S16 – Peak picked FT-IR spectrum of [2]rotaxane–polystyrene beads **8**.

IR Spectrum of [($C_{26}H_{28}NO_4$)Cr_7NiF_8($C_5H_7O_2$)$_{16}$] (10):

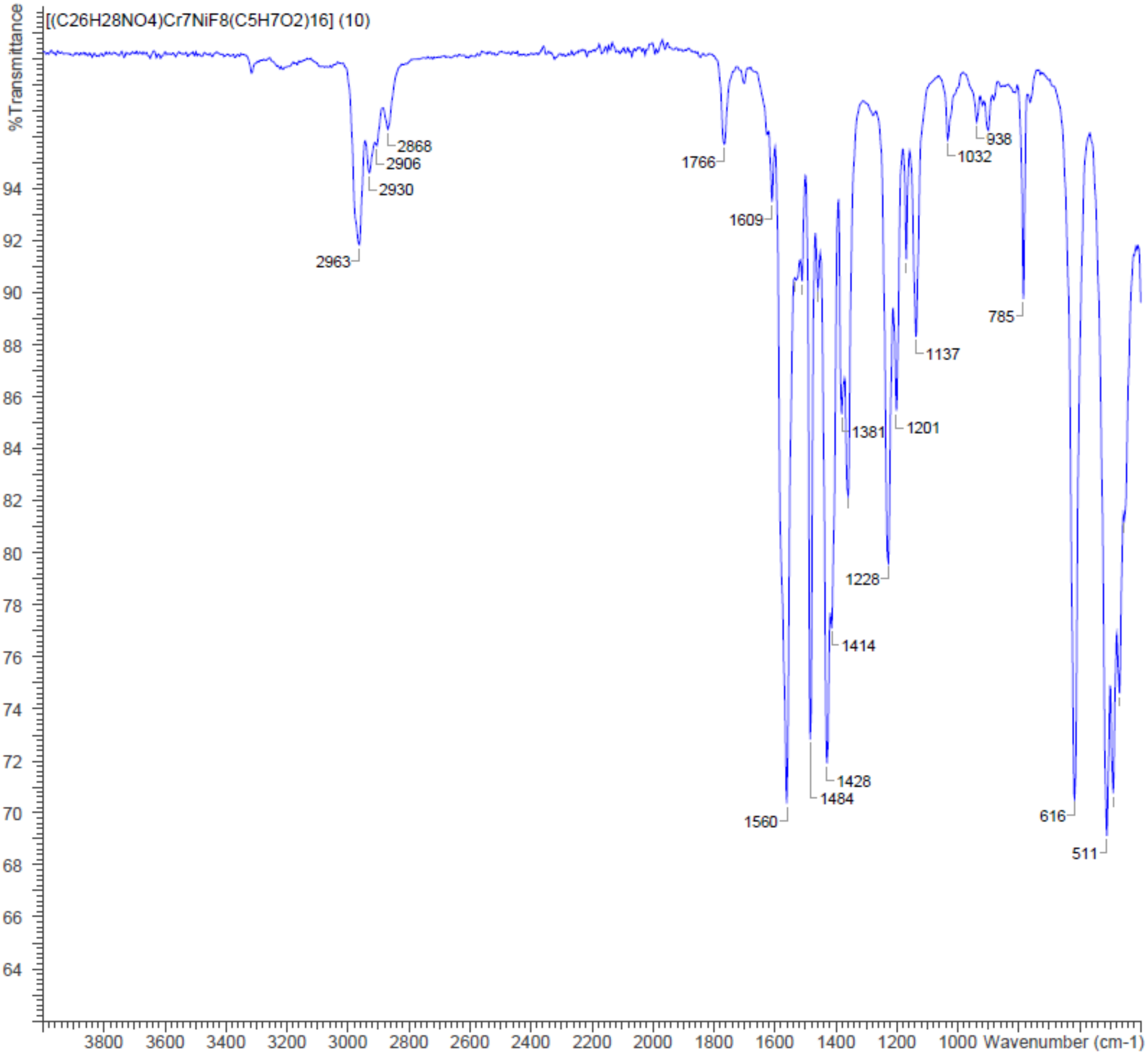

Figure S17 – Peak picked FT-IR spectrum of [($C_{26}H_{28}NO_4$)Cr_7NiF_8($C_5H_7O_2$)$_{16}$] **10**.

IR Spectrum of [2]rotaxane–polystyrene beads (11):

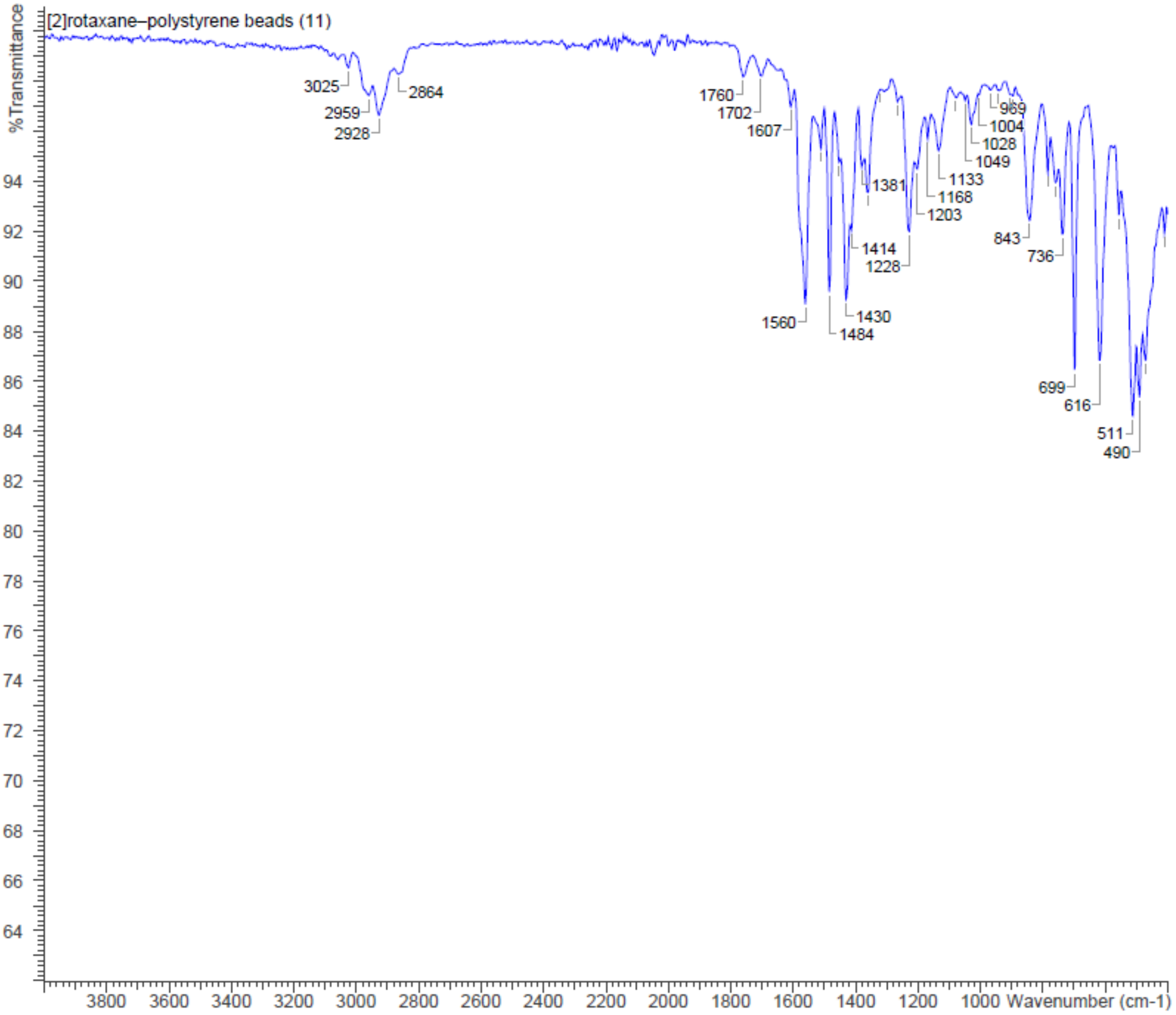

Figure S18 – Peak picked FT-IR spectrum of [2]rotaxane–polystyrene beads **11**.

S5. Supplementary methods: EPR measurements and spin counting experiment:

Continuous wave Q-band (~34 GHz) EPR spectra were recorded with a Bruker EMX580 spectrometer. The data were collected on polycrystalline powders and on solid polymeric beads at 280 K, and 5 K using liquid helium cooling. Spectral simulations were performed using the EasySpin 5.2.25 simulation software[7], simulation parameters can be found in table S2.

The spectra were simulated using a simple spin Hamiltonian incorporating only the individual *g*-matrices for the nitroxide signal and {Cr_7Ni} signal (where appropriate), and the nitroxide hyperfine interaction:

$$\hat{H} = u_B \hat{S}^{NO^{\cdot}} \cdot \boldsymbol{g}^{NO^{\cdot}} \cdot \boldsymbol{B} + u_B \hat{S}^{Cr7Ni} \cdot \boldsymbol{g}^{Cr7Ni} \cdot \boldsymbol{B} + \hat{S}^{NO^{\cdot}} \cdot \boldsymbol{A}^{NO^{\cdot}} \cdot \hat{I}^{NO^{\cdot}}$$

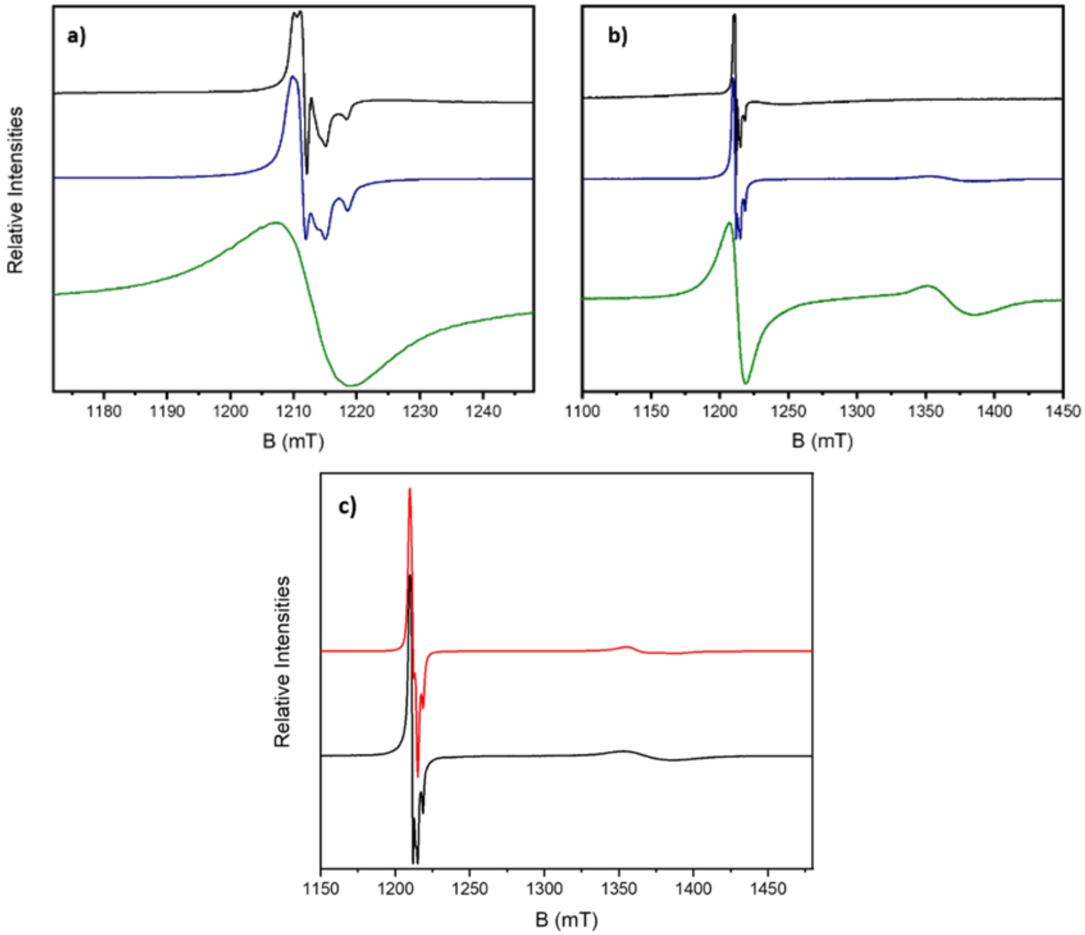

Figure S19: Q-Band (ca. 34 GHz) EPR spectra of **4** as a powder sample at 5 K (green), **7** (multiple bead samples) at 5 K (blue) and at 280 K (black). a) expansion of nitroxide section b) inclusion of {Cr_7Ni} signals, at high field resonances, c) simulation (red) for **7** (multiple bead samples) of experimental at 5K (black), frequency is 34.040610 GHz.

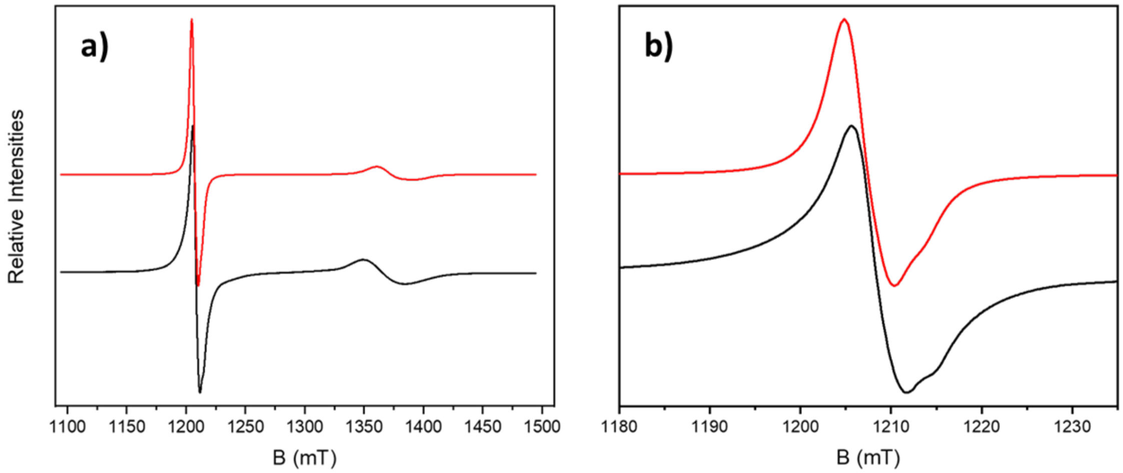

Figure S20: CW Q-Band (ca. 34 GHz) EPR spectra of a multiple bead sample at 5 K for **8** (black) and simulation (red), containing a) the nitroxide signal at low field and {Cr_7Ni} signal at higher field, b) enlarged section of the nitroxide signal. The experimental frequency is 33.932622 GHz.

Spin counting using EPR:

A single bead of **7** was measured (CW Q-Band, ca. 34 GHz) at 5 K and 280 K (Figure 2), the resulting spectra show a nitroxide signal at both temperatures, but the {Cr_7Ni} signal is only present at 5 K. The presence of a nitroxide signal at RT using just a single bead (albeit low S/N) presents an opportunity to quantify the number of absolute spins associated with a single bead, which equates to the number of rotaxanes bound to an individual bead.

Spin counting could be performed using the {Cr_7Ni} ring signal at 5 K. However, due to the sensitivity required to perform measurements even a *stable* 5 K fluctuates enough to influence the Boltzmann population (transition probability), thus affecting the signal intensities. As such each measurement would not produce the exact same conditions required for a set of spin counting experiments. Hence, all spin counting measurements and calibration samples here are all performed at RT using the nitroxide signal.

A calibration curve for spin counting was obtained by using two sets of calibration samples prepared from a stock solution of a 0.200 mM concentration of **4** in dry toluene. To try and provide a good coverage of the expected spin concentration of the beads, the stock solution was diluted to produce samples consisting of 0.10 mM, 0.090 mM, 0.080 mM, 0.070 mM, 0.060 mM, 0.050 mM, 0.040 mM, 0.030 mM, 0.020 mM, 0.010 mM and 0.005 mM. A single 2 mm (o.d.) Q-band quartz tube was used for all measurements and filled with 50 µL solution for each calibration measurement. The tube position was marked to ensure the height and orientation was kept constant throughout the calibration measurements, with the centre of the sample in line with the centre of the resonator cavity window. Experimental spectra were baseline corrected before integration. The resulting absorption spectra were then baseline corrected before a second integration to give the spectral intensity. The mean of the two calibration sets at each concentration was used for the final calibration curve.

Positioning requirements for bead measurements:

The majority of parameters that have an effect on the signal intensity can be kept constant, such as the equipment conditions; microwave power, modulation application and field sweep rates, as examples. However, the intensity of the EPR signal is also dependent on the coupling between the sample and resonator. This is defined as the sensitivity of the resonator, which is governed by the quality factor Q and the filling factor η.

The quality factor $Q = \nu_{res} / \Delta\nu$, where ν is the resonator frequency of the cavity and $\Delta\nu$ is the width at half the height of the resonance. The filling factor η is the ratio of the sample volume to the volume of the resonator, and a change in the filling factor can lead to a change in the Q factor. If the sample volume was to be increased (such as an addition of another bead) this would mean a greater interaction with the field B_1, leading to a larger η. This does provide an increase in signal. However, this can increase the width of the resonance $\Delta\nu$, leading to a drop in Q.[8] In order to adequately compare signal intensities between separate measurements, the quality factor and filling factor ideally need to be kept constant.

The calibration samples are a homogenous fluid solution; so as long as the same sample tube is used, the same volume of solution is present and the tube is in the same position in the resonator, the orientation should not influence the signal, keeping Q and η constant. Despite this during the calibration sample measurements the orientation of the tube was marked so the tube was put in exactly the same place each time. This was to keep everything constant as the orientation of the tube is very dependent for each bead measurement, a change in orientation would change the position of the beads in the cavity and thus influence the Q and η factors. One important note is, as each bead is not able to occupy the same position in space, each additional bead produces slightly different Q and η conditions to that of the previous bead(s) measurement. Though this is only a minor perturbation on the signal, it is unfortunately unavoidable for an experiment using solid beads. This highlights the importance of keeping the position of the sample within the resonator cavity the same for each measurement, if an accurate signal intensity and thus absolute spin counting experiment is to be achieved.

For measurement of the bead(s), an initial bead was inserted to the bottom of the EPR tube. Each additional bead was placed close to the previous one in order not to perturb its position. The tube was marked to ensure the height and orientation was kept constant throughout the bead measurements. The bottom of the tube was aligned with the centre of the resonator cavity window. The experimental spectra were worked up as per the calibration measurements.

The spin counting measurements were performed on 1 – 15 beads. Initially 1 bead was measured and then each further bead was added sequentially without perturbing the position of the previous beads. All experimental conditions and cavity tuning was kept constant throughout all of the measurements (including the calibration samples).

Spectra workup: After baseline correction, an initial integration was performed on each spectrum, resulting in an absorption like spectrum. A second baseline correction was performed before a second integration was applied to spectra, this results in a total signal intensity that can be applied to the calibration samples and a plot against them to find the absolute spin concentration for each bead sample.

The measurements for 2, 5, 10, 11 and 13 beads have been omitted due to an insufficient baseline correction, resulting in negative absorption sections that leads to a perturbation of the total signal intensity.

To find the number of absolute spins for the bead(s): A linear fit was applied to the calibration measurements, with the formula y = mx + c. The mean of the two calibration sets was calculated and the bead intensities then applied to give x, the data were then plotted to give absolute spin values.

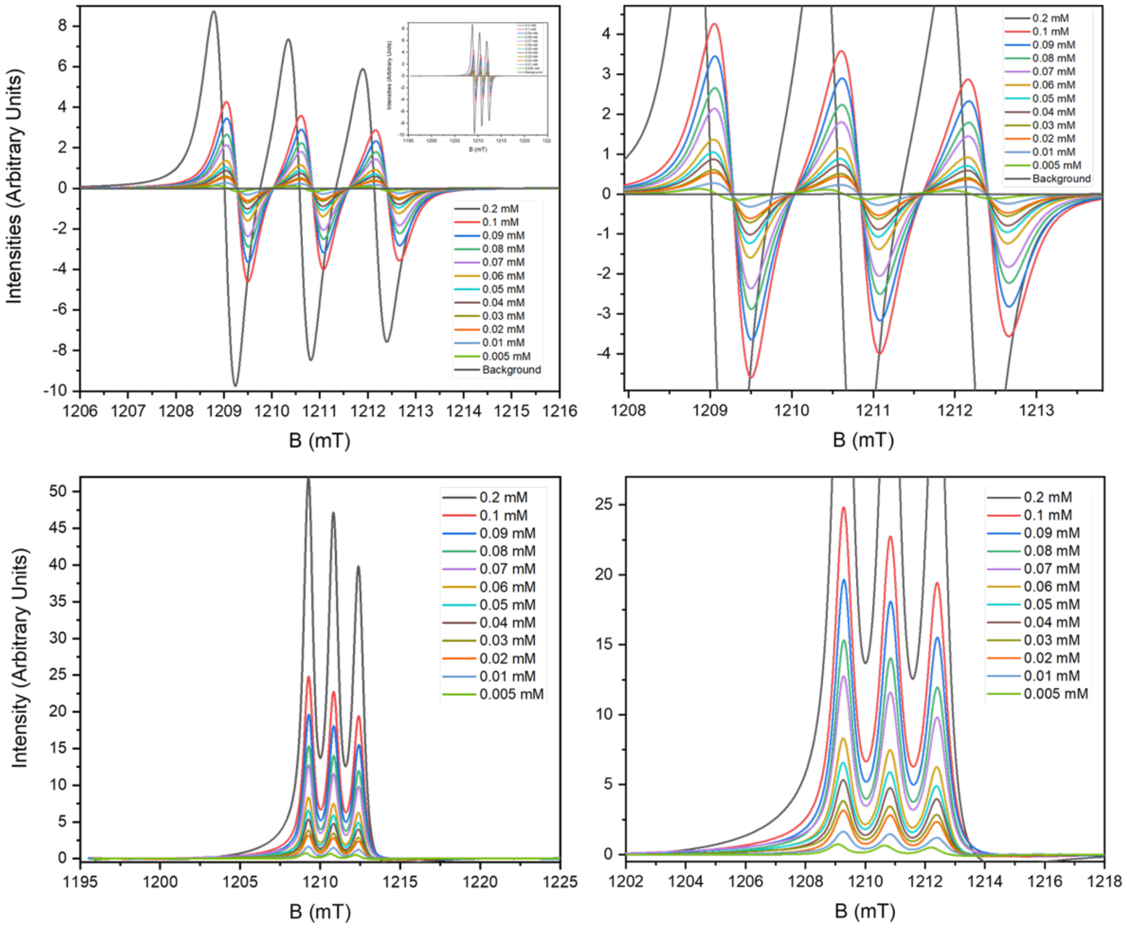

Figure S21: CW Q-Band (ca. 34 GHz) EPR spectra for calibration set-1 of '**4**' solution at RT. Top left: EPR spectra, with inset of full field width measured. Top right: Enlarged EPR spectra for 0.100 mM – 0.005 mM. Bottom left: EPR spectra after first integration. Bottom right: Enlarged EPR spectra after first integration of 0.100 – 0.005 mM. Concentration listed in figures.

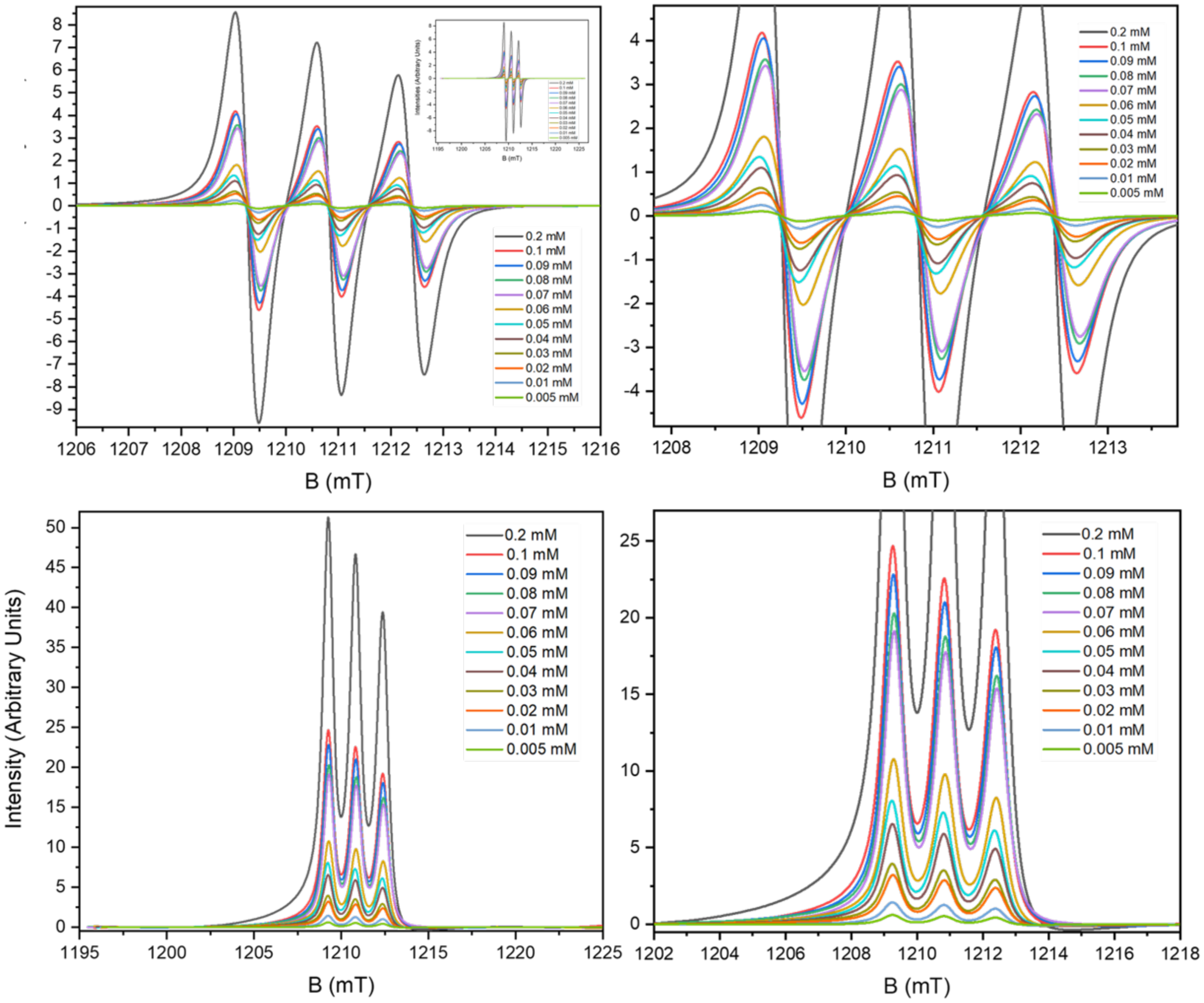

Figure S22: CW Q-Band (ca. 34 GHz) EPR spectra for calibration set-2 of **4** solution at RT. Top left: EPR spectra, with inset of full field width measured. Top right: Enlarged EPR spectra for 0.100 mM – 0.005 mM. Bottom left: EPR spectra after first integration. Bottom right: Enlarged EPR spectra after first integration of 0.100 – 0.005 mM. Concentration listed in figures.

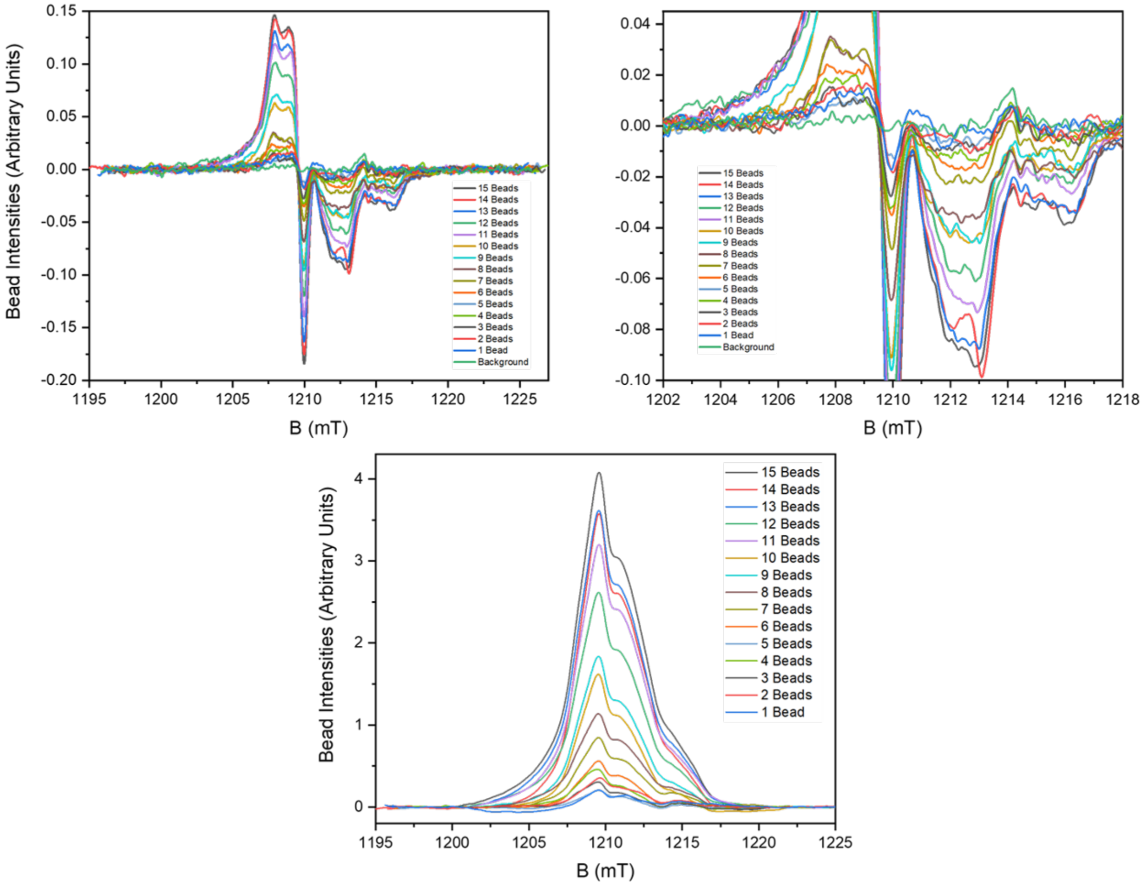

Figure S23: Q-band EPR spectra of **7** at RT. Top left: Full field width scan of the nitroxide signal. Top right: Enlarged spectra showing the lower intensity signals. Bottom: Spectra after first integration performed. Number of beads per samples listed in figures.

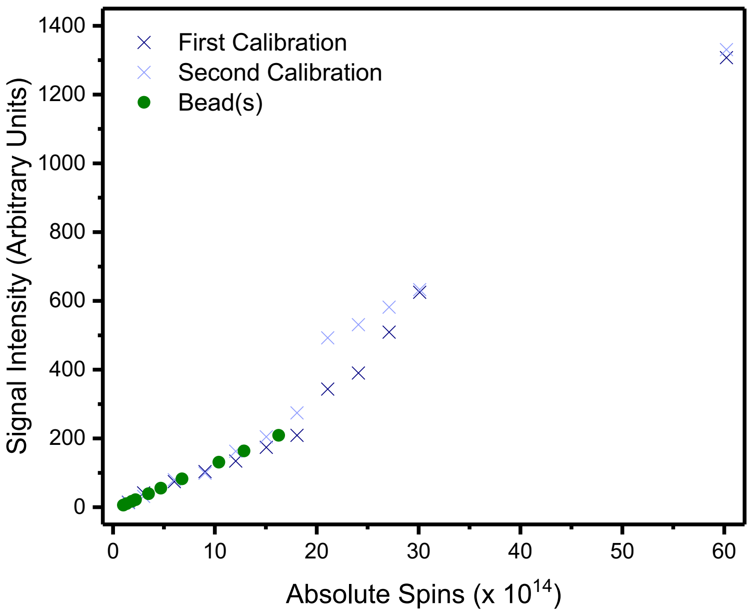

Figure S24: Bead intensities plotted against the mean gradient of the two calibration sets. From left to right the green circles represent the number of beads: 1, 3, 4, 6, 7, 8, 9, 12, 14, and 15 respectively.

S6. Supplementary References:

1 L. Bettinetti, S. Lober, H. Hubner and P. Gmeiner, *J. Comb. Chem*., 2005, **7**, 309-316.

2 S. Arseniyadis, A. Wagner and C. Mioskowski, *Tetrahedron Lett*., 2002, **43**, 9717–9719.

3 https://www.sigmaaldrich.com/GB/en/product/mm/855011.

4 (a) http://www.rapp-polymere.com/index.php?id=893&currency=968. (b) http://www.rapp-polymere.com/index.php?id=1219&currency=498.

5 (a) G. M. Sheldrick, *SADABS*, empirical absorption correction program based upon the method of Blessing. (b) L. Krause, R. Herbst-Irmer, G. M. Sheldrick and D. Stalke, An empirical correction for absorption anisotropy *J. Appl. Cryst.* **2015**, *48*. (c) R. H. Blessing, An empirical correction for absorption anisotropy, *Acta Crystallogr.* **1995**, *A51*, 33-38.

6 (a) Sheldrick. G. M. Crystal structure refinement with SHELXL, *Acta Crystallogr.*, 2015, *C71*, 3-8; (b) O. V. Dolomanov, L. J. Bourhis, R. J. Gildea, J. A. K. Howard and H. Puschmann, OLEX2: a complete structure solution, refinement and analysis program. *J. Appl. Cryst*., **2009,** *42*, 339–341

7 S. Stoll and A. Schweiger, *J. Magn. Reson.*, 2006, **178**, 42-55.

8 A. van der Est, Continuous-Wave EPR, *eMagRes*, 2016, **5**, 1411-1422.
