# Supplementary material for: Decorating polymer beads with 1014 inorganic-organic [2]rotaxanes as shown by spin counting
Source: Commun Chem. 2022 Jun 20;5:73. doi: 10.1038/s42004-022-00689-1 (PMC9814693; doi:10.1038/s42004-022-00689-1)
Supplement: Supplementary file 2 — Description of Additional Supplementary Files [file 42004_2022_689_MOESM2_ESM.pdf]

Description of Additional Supplementary Files

File name: Supplementary Data 1
Description: Crystallographic information files.
